# Novel 3-phenylquinazolin-2,4(1*H*,3*H*)-diones as dual VEGFR-2/c-Met-TK inhibitors: design, synthesis, and biological evaluation

**DOI:** 10.1038/s41598-023-45687-y

**Published:** 2023-10-30

**Authors:** Abdelfattah Hassan, Ahmed M. Mosallam, Amal O. A. Ibrahim, Mohamed Badr, Aboubakr H. Abdelmonsef

**Affiliations:** 1 Department of Medicinal Chemistry, Faculty of Pharmacy, South Valley University, Qena, Egypt; 2 Department of Chemistry, Faculty of Science, South Valley University, Qena, Egypt; 3 Department of Biochemistry, Faculty of Pharmacy, Menoufia University, Menoufia, Egypt

**Keywords:** Cancer, Chemical biology, Computational biology and bioinformatics, Chemistry

## Abstract

Multitarget anticancer drugs are more superior than single target drugs regarding patient compliance, drug adverse effects, drug-drug interactions, drug resistance as well as pharmaceutical industry economics. Dysregulation of both VEGFR-2 and c-Met tyrosine kinases (TKs) could result in development and progression of different human cancers. Herein, we reported a novel series of 3-phenylquinazolin-2,4(1*H*,3*H*)-diones with thiourea moiety as dual VEGFR-2/c-Met TKs. Compared to sorafenib, cabozantinib went behind VEGFR-2 inhibition to target c-Met TK. The dual VEGFR-2/c-Met inhibitory activity of cabozantinib is due to a longer HB domain than that of sorafenib. Based on pharmacophore of cabozantinib analogues, we designed new dual VEGFR-2/c-Met TKs. We synthesized the target compounds via a new single pot three-component reaction. The cytotoxic activity of synthesized compounds was conducted against HCT-116 colorectal cancer cell line. Compounds **3c** and **3e** exhibited the highest cytotoxic activity against HCT-116 cell line (IC_50_ 1.184 and 3.403 µM, respectively). The in vitro enzyme inhibitory activity was carried out against both VEGFR-2 and c-Met TKs. Compound **3e** has the highest inhibitory activity against both VEGFR-2/c-Met (IC_50_ = 83 and 48 nM, respectively). Docking studies showed that α-oxo moiety in quinazoline ring formed hydrogen bond HB with Met1160 residue in the adenine region of c-Met TK.

## Introduction

The most serious health challenge of humanity is cancer^1^. It was predicted that about 30 million people will be diagnosed as new cancer patients by 2040^1^. As a result, identifying novel drug targets and developing more selective chemotherapeutic agents are important goals of current drug research^2^. Although recently developed single target drugs are more selective and safer than awkward conventional anticancer therapeutics, they can probably develop drug resistance^3,4^. One way to solve this issue is a combination therapy but this can increase undesired side effects and toxicity^5^. In contrast, several studies proved that multitarget small molecules can overcome this resistance with acceptable safety profile^3,4^. Compared to combination therapy, multitarget drugs enhance patient compliance, void detrimental off-target effects, decrease drug-drug interactions, and save excessive manufacturing costs^5^.

There are over five hundred protein kinases in our bodies representing the most abundant family amidst all function proteins. Protein kinases harmonically control vital cellular processes like cell division, proliferation, metabolism, migration, and apoptosis^6^. Protein kinases deregulation is responsible for a number of fatal illnesses, including cancer^2^. Accordingly, the inhibition of these deregulated protein kinases is a viable strategy to combat cancer^6^.

Binding of vascular endothelial growth factor (VEGF) and hepatocyte growth factor (HGF) to their receptors VEGFR-2 and c-Met, respectively results in conformational changes and dimerization of the receptors followed by phosphorylation of multiple tyrosine residues in the intracellular domain^2^. Consequently, a cascade of intracellular signaling pathways were sparked off^2^.

Dysregulated VEGFR-2 and c-Met tyrosine kinases (TKs) work together to promote angiogenesis that result in development and progression of different human cancers^7^. Both tumor growth and metastasis are depend mainly on angiogenesis which is controlled by VEGFR-2 and c-Met signaling^8^. Consequently, multitarget molecules that can inhibit both VEGFR-2 and c-Met simultaneously may be more effective than single target molecules since they can shut down many signaling pathways implicated in tumor angiogenesis, proliferation, and metastasis^7,9^. Additionally, drug resistance is more common with single target drugs than multitarget ones^9^.

## Rationale and molecular design

**Figure 1:**
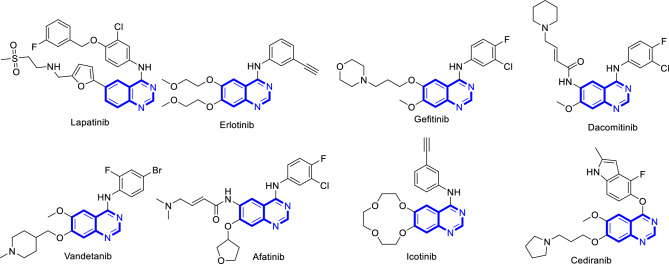
Clinically used quinazoline containing TK inhibitors.

There are four pharmacophoric elements required for type II dual VEGFR-2/c-Met inhibitors namely nitrogenous heterocycle, linker, hydrogen bonding (HB) domain and hydrophobic moiety^9^. The first is a planar nitrogenous heterocyclic system (in blue) mainly six-membered monocyclic rings like pyridine^10–13^, pyrimidine^7,10,14^, and their fused benzo rings like quinoline^10,12^, quinazoline^9,10,12,15^, as well as fused hetero- rings like thienopyridine^12,16^, thienopyrimidine^2^, pyrrolopyridine^17,18^, pyrrolopyrimidine^18^, triazolopyrazine^3^, pyrrolotriazine^12,19^. Quinazoline ring is incorporated in several FDA approved 4-anilinoquinazoline EGFR inhibitors like lapatinib, erlotinib, gefitinib, dacomitinib, vandetanib, afatinib and icotinib as well as an VEGFR-2 inhibitor, cediranib (Fig. 1)^12,20^.

**Figure 2:**
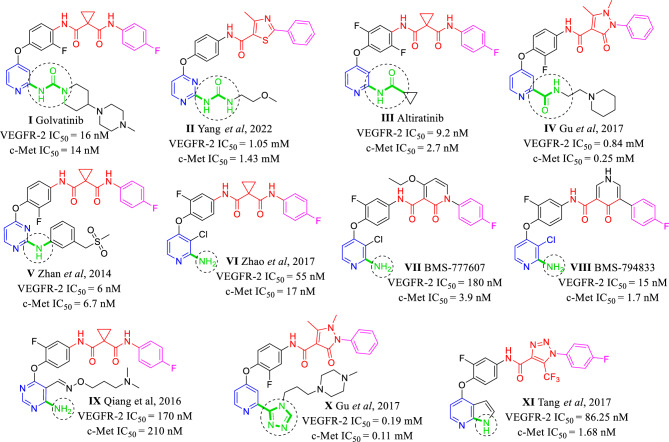
Some reported dual VEGFR-2/c-Met inhibitors with α-HB group (in green).

The heterocyclic ring occupies the hinge region of both TKs and forms HB via its nitrogen with the highly conserved Cys919 and Met1160 residues in VEGFR-2 and c-Met proteins, respectively. It is worth mentioning that several dual VEGFR-2/c-Met inhibitors contain HB group at α-position of the nitrogenous heterocycle that augment binding of inhibitor to the adenine region of the protein through forming additional HBs (Fig. 2). This α-HB group may be amino^7,21^, oxo^9,22^, amido^19^, carbamoyl^11^, triazolyl^10,11^, ureido^10,19^, or even the pyrrole nitrogen of pyrrolopyridine system^23^. In addition to HB of pyrimidine nitrogen with Cys919 and Met1160 in the adenine pocket of VEGFR-2 and c-Met TK, respectively, α-ureido group of compound **II** formed extra two HB with Cys919 in the hinge region of VEGFR-2 TK. Moreover, compound **II** formed HB with Met1160 in the hinge region of c-Met TK^10^. Carboxamido group of altiratinib **III** formed extra HB with Met1160 in the hinge region of c-Met TK^24^. α-Amino group in compound **V** act as hydrogen bond donor (HBD) forming HB with Met1160 in the hinge region of c-Met TK. Contrary, the *N*-methylated analogue of compound **V** showed no activity against both VEGFR-2 and c-Met TK^7^. The authors purposed that there is a relationship between HB of the α-amino with the adenine region of TKs and the inhibitory activity of the compound **V**^7^. α-Amino group of **VII BMS-777607** act as HBD forming HB with Met1160 in the hinge region of c-Met TK^25^. Amino group of compound **IX** forms extra HB with Cys919 and Met1160 in the hinge region of VEGFR-2 and c-Met TKs, respectively^14^. Triazole ring of compound **X** formed extra HB with Cys919 in the hinge region of VEGFR-2 TK. In addition, It also form new HB with Tyr1159 in adenine pocket of c-Met TK^11^. Tang et al., synthesized several series of pyrrolo[2,3-b]pyridine c-Met inhibitors with considerable VEGFR-2 inhibitory activity^23,26^. Nitrogen of pyrrole ring in **XI** act as HBD forming HB with Met1160 in the hinge region of c-Met TK^26^.

**Figure 3:**
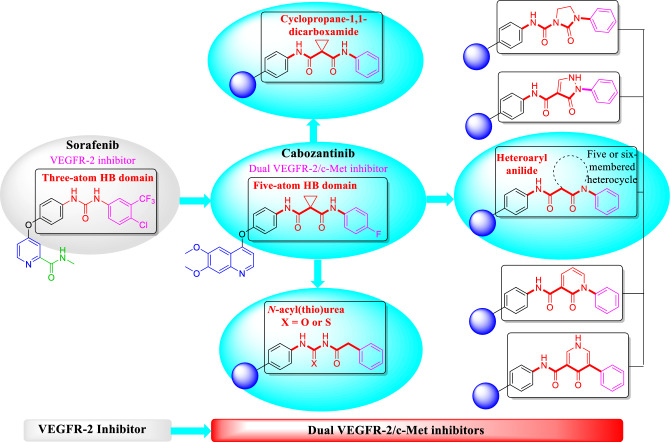
HB domain requirements for dual VEGFR-2/c-Met TKs inhibition over VEGFR-2 single inhibition.

**Figure 4:**
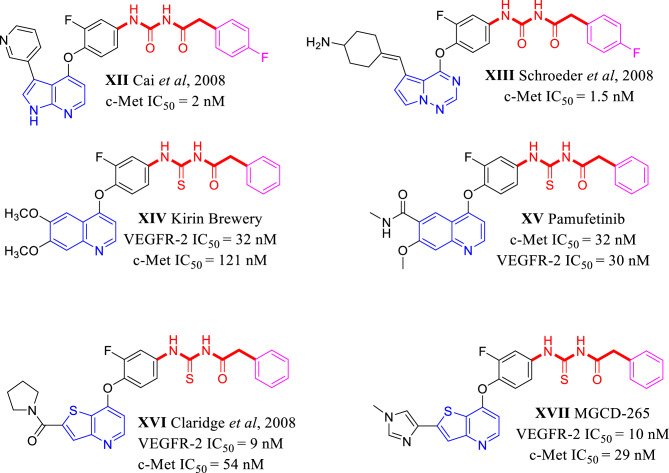
Some reported *N*-acyl(thio)urea containing c-Met and dual VEGFR-2/c-Met inhibitors.

The second pharmacophoric element is a hydrophobic linker (in black) which connects nitrogenous heterocycle and HB domain. This liker has little importance in protein binding, but it puts other pharmacophoric elements in their correct position and orientations^27,28^. The third is a HB domain (in red) which forms HB with both Asp1046 and Glu885 of the VEGFR-2 TK as well as both Asp1222 and Lys1110 of the c-Met TK. In the type II TKIs, the main difference between VEGFR-2 inhibitors and dual VEGFR-2/c-Met inhibitors is the relatively longer HB domain of dual inhibitors^7,12^. VEGFR-2 inhibitors contain 2–3 atom HB domain like carboxamide (e.g., sunitinib and nintedanib) or urea (e.g., sorafenib, regorafenib and lenvatinib)^28^. On the other hand, the dual VEGFR-2/c-Met inhibition is originated from the presence of an HB domain with 4 or more atoms^7,12^. HB domain of dual VEGFR-2/c-Met is usually a dicarboxamide structure which can be a widespread cyclopropane-1,1-dicarboxamide like clinically used cabozantinib and foretinib as well as hundreds of other investigated compounds^2,4,12,14,15,19,24^. The dicarboxamide structure can be also involved in heteroaromatic anilides like *N*-phenyl-2-oxoimidazolidine-1-carboxamide^29^, *N*-phenyl-3-oxo-2,3-dihydro-1*H*-pyrazole-4-carboxamide (e.g., compound **IV**)^4,11^, *N*-phenyl-2-oxo-1,2-dihydropyridine-3-carboxamide (e.g., **VII** BMS-777607 and merestinib)^3,25^, *N*-phenyl-4-oxo-1,4-dihydropyridine-3-carboxamide (e.g., **VIII** BMS-794833) (Fig. 3)^18^. Finally, *N*-acyl(thio)urea can surrogate a dicarboxamide structure and exhibited a dual VEGFR-2/c-Met inhibitory activity (Fig. 4)^16,30–34^. The forth is a hydrophobic moiety (in purple) that occupies allosteric pocket of the TKs^3,4,6,35^.

**Figure 5:**
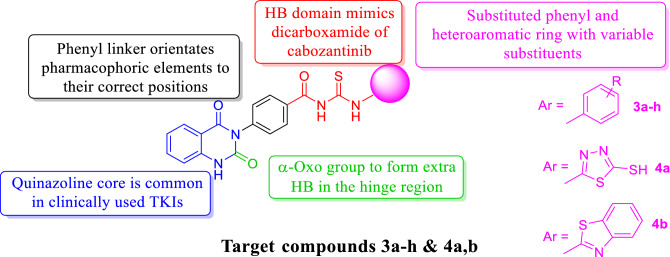
Design of new quinazoline dual VEGFR-2/c-Met inhibitors.

Consequently, cabozantinib was selected as a lead compound, building up the pharmacophore for dual VEGFR-2/c-Met inhibition through incorporation of *N*-acylthiourea moiety as HB domain that mimics dicarboxamide structure of cabozantinib Fig. 5^3^. Quinazolin-2,4(1*H*,3*H*)-dione ring was chosen to occupy adenine pocket of the TKs. α-Oxo moiety was involved in several reported TKIs like dovitinib, nintedanib, orantinib, and sunitinib^36^. Therefore, α-oxo moiety of target compounds was designed to augment HB and increase the affinity for both the VEGFR-2 and c-Met TKs. Finally, we different phenyl and heteroaryl rings with variable substituents were designed to occupy allosteric pocket.

## Result and discussion

### Chemistry

**Scheme 1:**
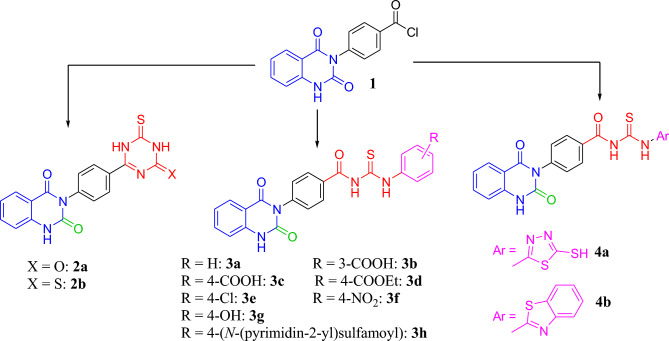
Synthetic routes of 3-phenylquinazolin-2,4(1*H*,3*H*)-dione derivatives **2a,b**, **3a-h** and **4a,b**. Reagent and conditions: NH_4_SCN, (thio)urea or Ar-NH_2_, TEA, dioxane, reflux.

The synthetic pathway of 3-phenylquinazolin-2,4(1*H*,3*H*)-dione derivatives **2a,b**, **3a-h** and **4a,b** is depicted in Scheme 1^37,38^. The first step involves the synthesis of intermediate benzoyl isothiocyanate by the reaction of 4-(2,4-dioxo-1,4-dihydro-2*H*-quinazolin-3-yl)-benzoyl chloride **1** and ammonium thiocyanate under reflux. In the second step, the latter intermediate got readily transformed to the final products by nucleophilic attack of the amino groups of various used reagents such as urea, thiourea, aromatic and hetero amines via a single pot three-component reaction.

The benzoyl isothiocyanate has been employed in synthesis of some new six membered heterocyclic skeletons like thioxo-triazin-2-one and triazine-2,4-dithione, by nucleophilic addition with urea and thiourea, respectively. Herein, an equimolar reaction of benzoyl isothiocyanate with urea and/or thiourea under reflux for 6–8 h, yields an intermediate which underwent cyclodehydration to furnish compounds **2a,b**, respectively. The success of cyclization by water elimination was supported by spectral data of the resulting triazines **2a,b**. Their IR spectra showed absorption bands at (3406, 3226), (1716, 1655) and 1237 cm^−1^ for NH’s, C=O’s, and C=S, respectively. The ^1^H-NMR spectrum of triazine **2a** exhibited their presence of two tautomeric forms from the downfield signals at 7.98, and 8.10 ppm for the NH protons. In the same manner, ^1^H-NMR of triazine **2b** represented 8.06 and 8.08 for NH protons.

The formation of the afforded *N*-benzoyl-*N*′-phenylthiourea derivatives **3a-h** was carried out by the nucleophilic reaction of different aromatic amines with benzoyl isothiocyanate in dioxane and few drops of TEA for 6–10 h. Their chemical structures were supported by IR and ^1^H-NMR spectra. For example, the IR bands of compound **3a** illustrated the presence of NH, C=O’s and C=S at 3243, 1718, 1663 and 1268, respectively. In addition, ^1^H-NMR spectrum of compound **3a** exhibited three singlet signals for NH protons at 12.63, 11.72, and 11.66 ppm, respectively, along with aromatic protons at 7.26–8.11 ppm.

Similarly, the intermediate benzoyl isothiocyanate reacts with some heterocyclic amines namely 5-amino-1,3,4-thiadiazole-2-thiol and 2-aminobenzothiazole under reflux for 10–12 h to furnish thiadiazole and benzothiazole **4a,b**, respectively.

### Biology

#### In vitro antiproliferative activity against HCT-116

HCT-116 colorectal cancer cell line is characterized by overexpression of both VEGFR-2 and c-Met TKs. Consequently, we selected it to study the cytotoxic activity of the target derivatives^9^. We tested the effect of several concentrations of the designed derivatives on HCT-116 cells by using MTT assay. Table 1 represents the IC_50_ of all synthesized derivatives as well as that of the reference drug, cabozantinib.

**Table 1:** In vitro cytotoxicity of compounds **2a,b**, **3a**–**h**, **4a,b,** and cabozantinib against HCT116 cell line.

Compound	IC_50_ µM	Compound	IC_50_ µM
**2a**	27.030 ± 1.43	**3e**	3.403 ± 0.18
**2b**	8.808 ± 0.47	**3f**	113.500 ± 6.00
**3a**	9.379 ± 0.50	**3g**	47.020 ± 2.49
**3b**	53.390 ± 2.82	**3h**	29.820 ± 1.58
**3c**	1.184 ± 0.06	**4a**	19.900 ± 1.05
**3d**	2.243 ± 0.12	**4b**	20.270 ± 1.07
Cabozantinib	16.350 ± 0.86		

The designed compounds showed noticeable antiproliferative activities. 5 out of 12 derivatives namely, **2b**, **3a**, **3c**,** 3d**, and **3e** exhibited noteworthy cytotoxic activity (IC_50_ = 1.184–9.379 µM) that was more superior than that of the positive reference, cabozantinib. Additionally, compounds **4a** and **4b** (IC_50_ = 19.90 and 20.27 µM, respectively) displayed comparable cytotoxic activities to cabozantinib. The rest of target compounds, **2a**, **3b**, **3g**, and **3h** exhibited acceptable cytotoxic activity (IC_50_ = 27.030–53.39 µM). Compound **3f** showed the least cytotoxic activity (IC_50_ = 113.500 µM). Compound **3c** (with *p*-carboxyl moiety) represented higher cytotoxic activity than compound **3b** (with *m*-carboxyl moiety) that suggests the regioisomerism effect on cytotoxic activity with *p*-substitution is superior to *m*-substitution. Apart from **3f**, compounds with electron withdrawing group on phenyl ring represented higher cytotoxic activity as compared to unsubstituted phenyl.

#### In vitro toxicity against normal cells

The cytotoxic activity of selected two compounds namely, **3c** and **3e** against WI38 normal cell line was evaluated to study the safety of the designed compounds to the normal cell (Table 2). Although, both tested compounds exhibited higher cytotoxicity against normal cells than cabozantinib, they are better in terms of selectivity index. Compound **3c** showed 20 times selective cytotoxicity against the colorectal cancer cells over the normal cells. Moreover, compound **3e** showed more than 3 times selective cytotoxicity against the colorectal cancer cells over the normal cells. Compounds **3c** and **3e** as well as cabozantinib exhibited considerable safety as the values of SI of all of them are more than 2^39^.

**Table 2:** In vitro cytotoxicity of compounds **3c** and **3e** against WI38 normal cell line.

Compound	IC_50_ µM	Selectivity index
**3c**	23.76 ± 1.41	20.07
**3e**	11.61 ± 0.69	3.41
Cabozantinib	44.71 ± 2.65	2.73

#### In vitro activity against c-Met and VEGFR-2 tyrosine kinases

Based on our rationale design of dual VEGFR-2/c-Met inhibition, we selected the most active cytotoxic derivatives to the HCT-116 cancer cells for scrutinization of their inhibition activity against both VEGFR-2 and c-Met TKs. Table 3 represents the inhibition activity of tested derivatives as well as cabozantinib. All tested derivatives showed acceptable inhibitory activity against the target TKs. Compound **3d** exhibited comparable activity to cabozantinib against VEGFR-2 enzyme with (IC_50_ = 51 nM). In addition, compound **3e** showed noticeable inhibitory activity against VEGFR-2 enzyme with (IC_50_ = 83 nM). Regarding the c-Met TK, both compounds **3c** and **3e** represented inhibition activity against c-Met TK with IC_50_ values 74 and 48 nM, respectively.

**Table 3:** Inhibitory activity of compounds **2b**, and **3c-e** against VEGFR-2 and c-Met TKs.

Compound	VEGFR-2 IC_50_ nM	c-Met IC_50_ nM
**2b**	263 ± 15	141 ± 8
**3c**	138 ± 8	74 ± 4
**3d**	51 ± 3	442 ± 26
**3e**	83 ± 5	48 ± 3
Cabozantinib	59 ± 3	30 ± 2

#### Apoptosis assay

**Figure 6:**
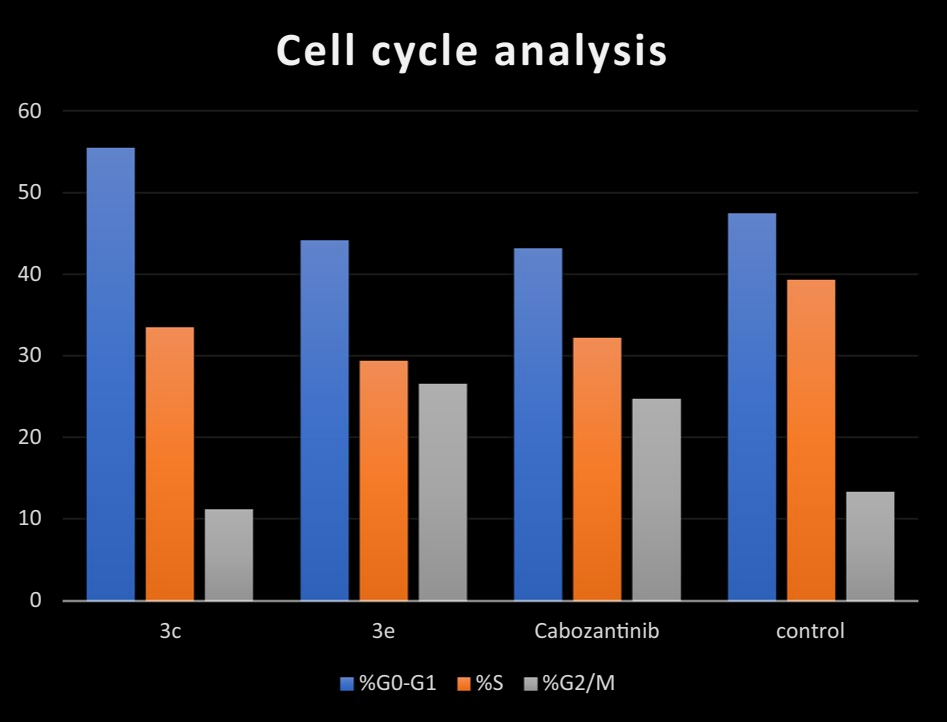
Flow cytometric analysis of cell cycle phases after treatment with **3c**, **3e** and cabozantinib.

To go deeper in the mechanism of designed derivatives, the cell cycle arrest study of compounds **3c** and **3e** was conducted by using Annexin V-FITC/PI staining (Table 4 and Fig. 6). HCT-116 cells were treated by cabozantinib and the two tested compounds at their IC50 concentrations. Compared with the control group, HCT-116 cell cycle was blocked in G0/G1 phase after treatment with **3c** (55.41%). On another hand, compound **3e** (26.51%) had a higher ability to enhance the population of HCT-116 cells in G2/M process than cabozantinib (24.72%).

**Table 4:** Cell cycle analysis in HCT-116 colon cancer cell line treated with compounds **3c** and 3**e**.

Compound	%G0–G1	%S	%G2/M
**3c**	55.41	33.46	11.13
**3e**	44.13	29.36	26.51
Cabozantinib	43.12	32.16	24.72
Control	47.41	39.28	13.31

**Figure 7:**
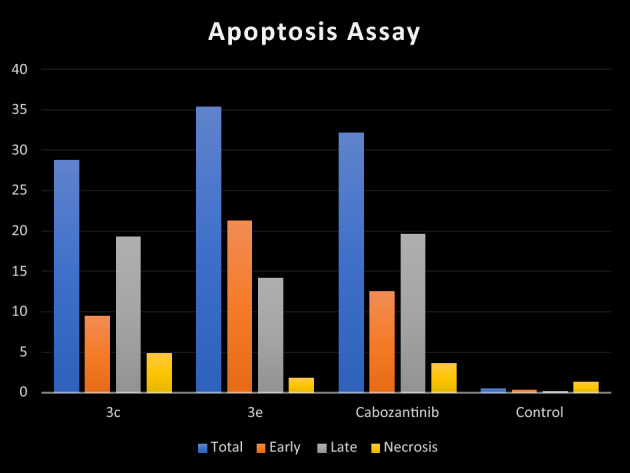
Effect of **3c**, **3e** and cabozantinib on HCT-116 cells in annexin V-FITC staining test.

**Figure 8:**
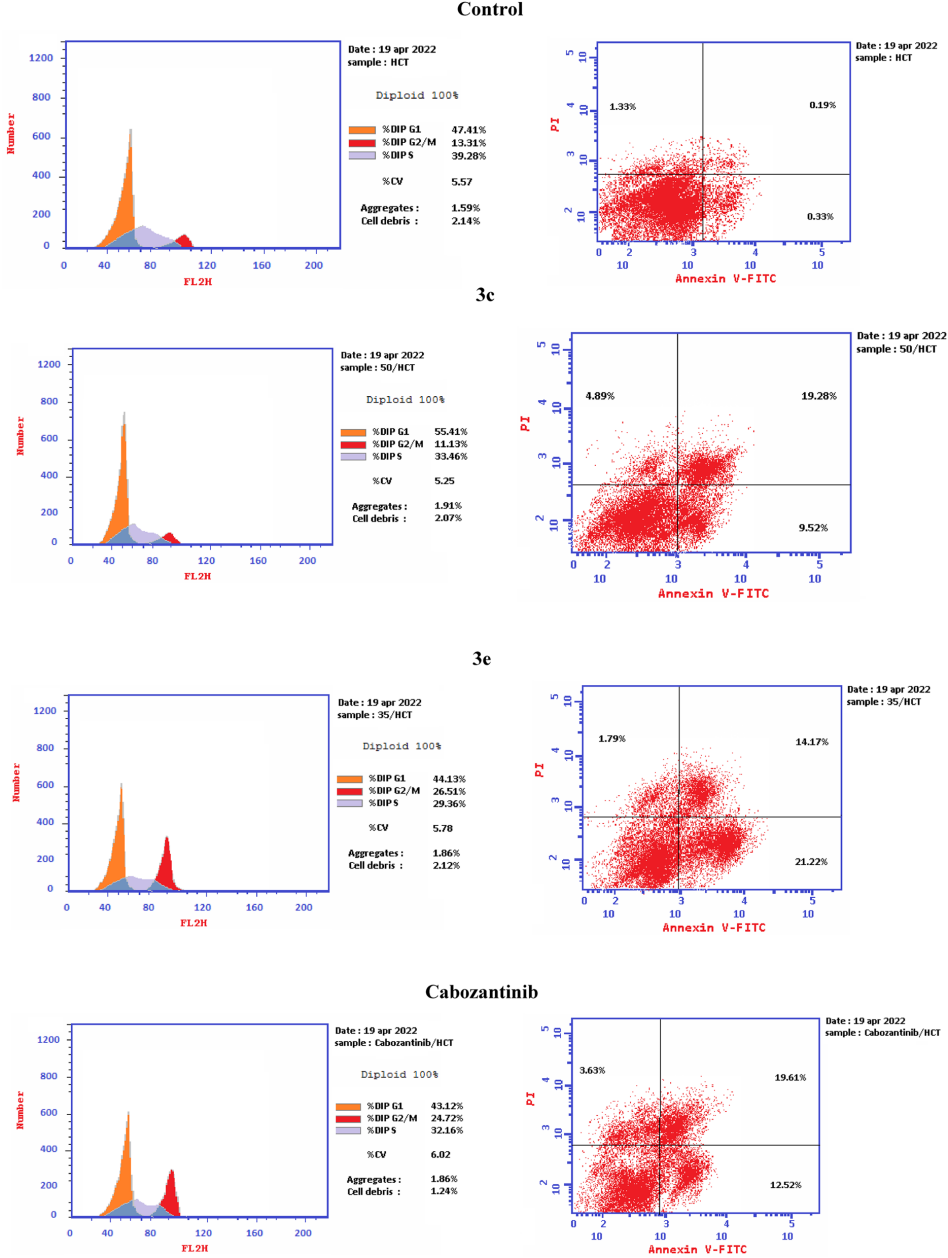
Representative cytograms of apoptotic HCT-116 cells induced by **3c** and **3e** compared to cabozantinib for 24 h.

Moreover, we studied the apoptosis mechanism of HCT-116 that was induced by tested derivatives **3c** and **3e** as well as cabozantinib (Table 5 and Fig. 7). There was an increase in the number of both early and late apoptotic cells of both tested compounds **3c** and **3e**. The number of late apoptotic cells of **3e** (35.39%) was higher than that of reference drug, cabozantinib (32.13%). Also, the number of late apoptotic cells of **3c** (19.28%) was approximately equal to that of cabozantinib (19.61%). Totally, both designed compounds **3c** and **3e** were able to induce remarkable apoptosis. Moreover, compound **3e** succeeded in apoptosis induction more efficiently than cabozantinib (Fig. 8).

**Table 5:** Apoptosis induction analysis for compounds **3c**,** 3e** and cabozantinib.

Compound	%Apoptosis			%Necrosis
	%Total	%Early	%Late	
**3c**	28.8	9.52	19.28	4.89
**3e**	35.39	21.22	14.17	1.79
Cabozantinib	32.13	12.52	19.61	3.63
Control	0.52	0.33	0.19	1.33

### In silico studies

#### Molecular modeling studies

**Figure 9:**
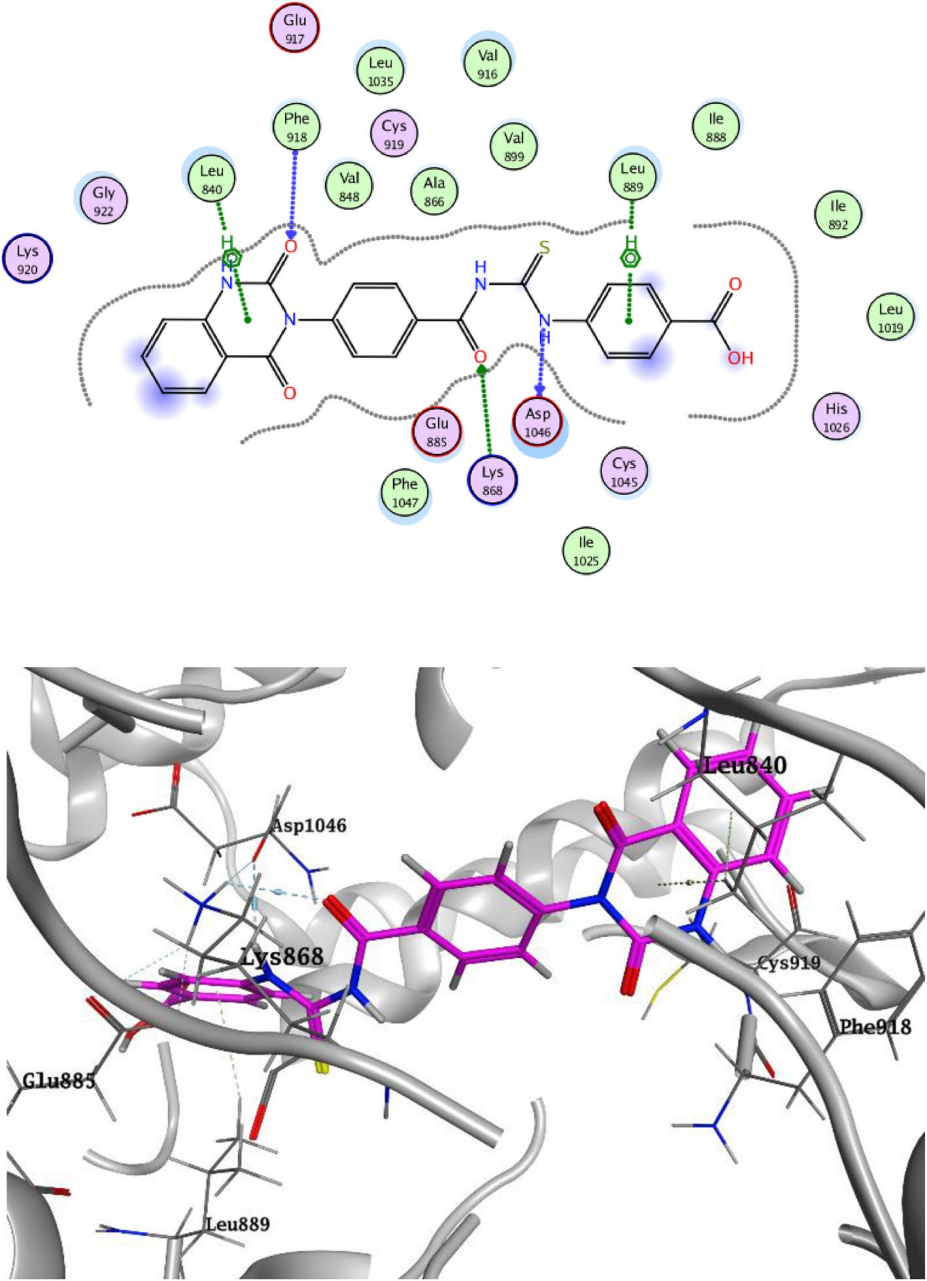
Docking style of compound **3c** with VEGFR-2 TK (PDB: 4ASD).

**Figure 10:**
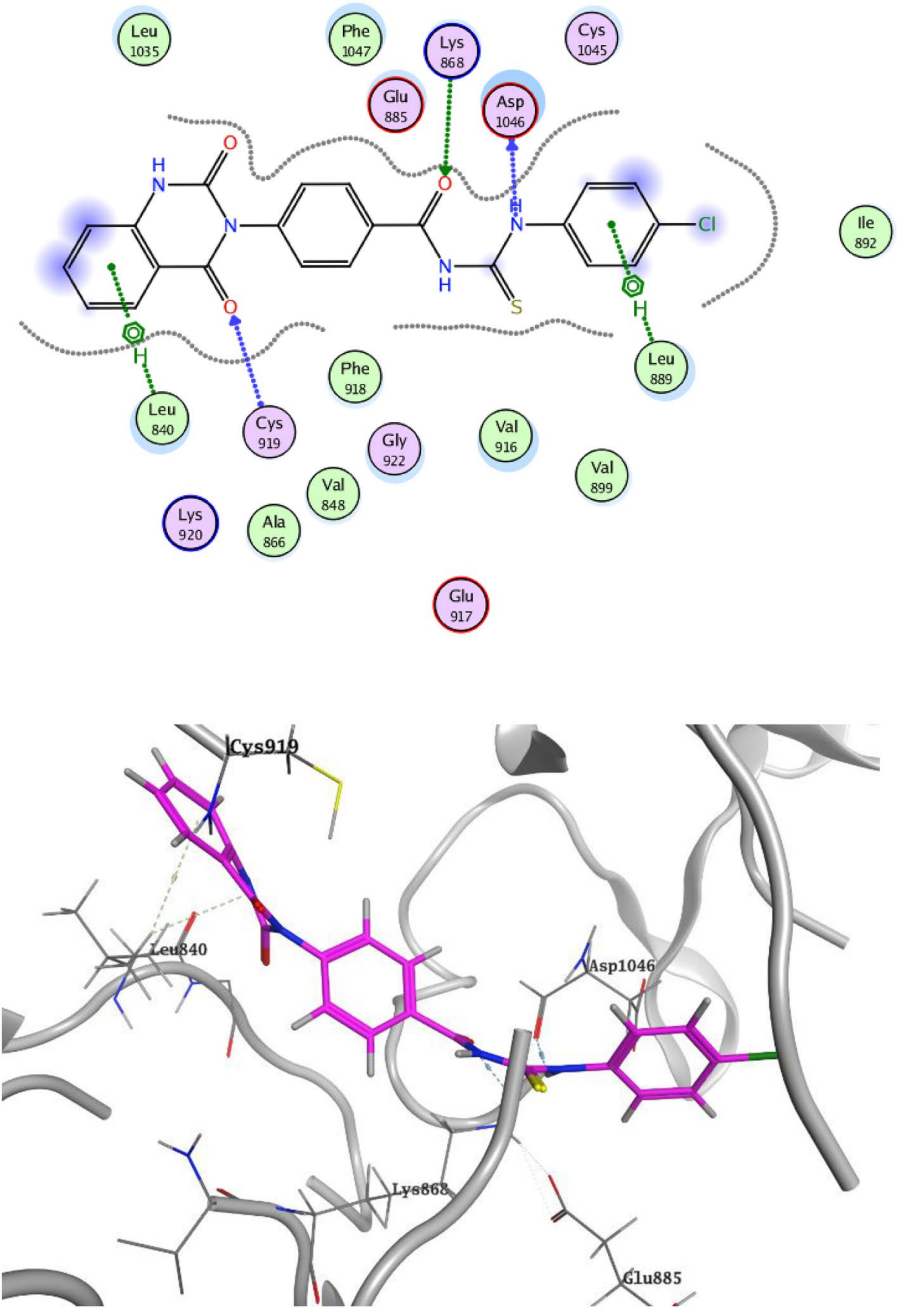
Docking style of compound **3e** with VEGFR-2 TK (PDB: 4ASD).

Molecular docking of the target compounds was carried out in the active site of c- VEGFR-2 (PDB: 4ASD) and c-Met (PDB: 3LQ8) TKs. Docking styles of compounds **3c** and **3e** in the active site of VEGFR-2 TK are shown in Figs. 9 and 10, respectively. The thiourea group of compound **3c** showed HB with a highly conserved residue Asp1046 in the HB region and α-oxo group of quinazoline formed HB with backbone amide of Phe918 in the hinge region. Moreover, compound **3c** exhibited hydrophobic interactions with both Leu840 and HB with Lys868. 4-Oxo moiety of compound **3e** declared HB with the conserved residue Cys919 in the hinge region. Compound **3e** showed another two HB with Asp1046 and Lys868 in addition to hydrophobic interaction with Leu840^27,28^.

**Figure 11:**
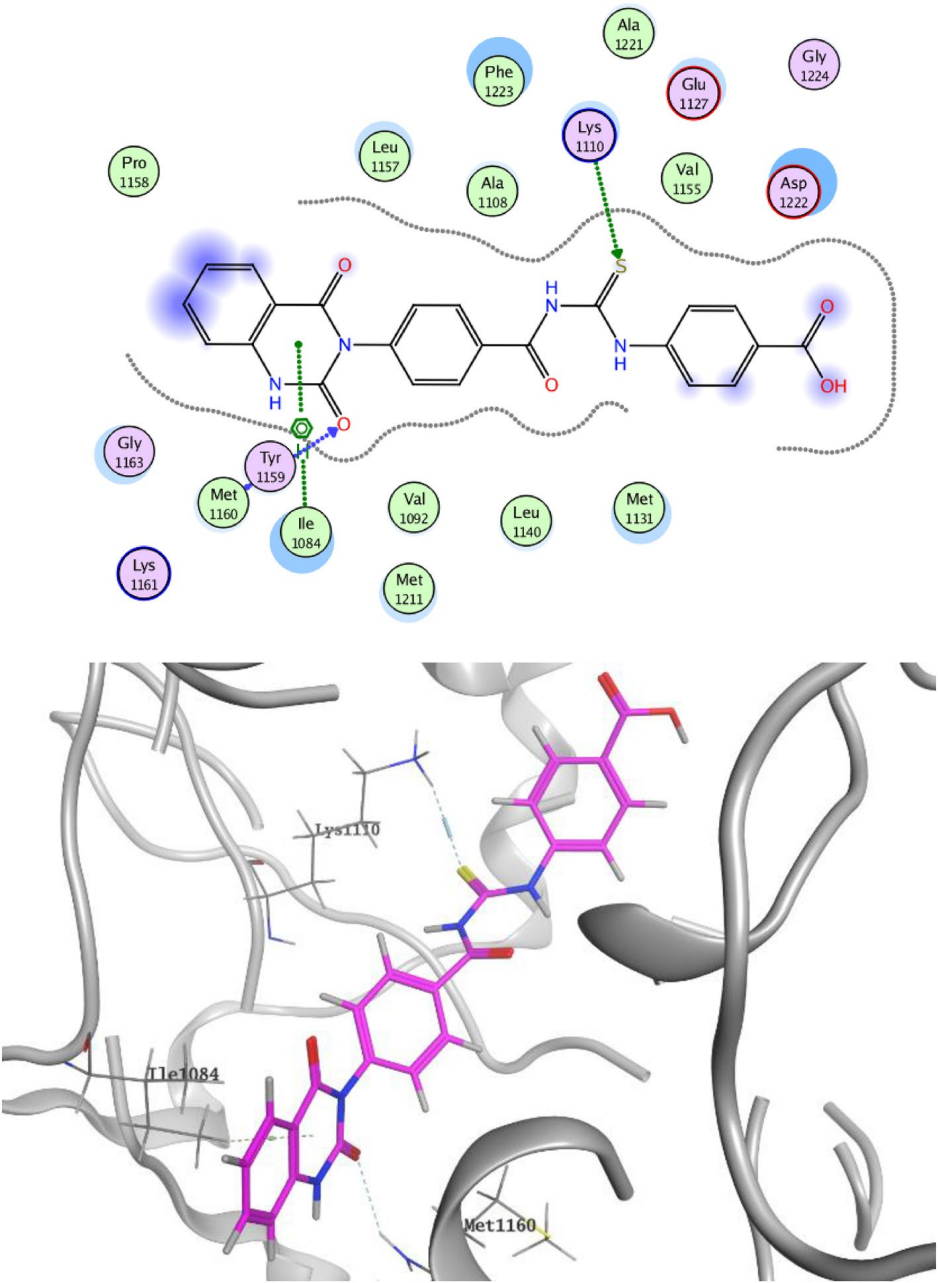
Docking style of compound **3c** with c-Met TK (PDB: 3LQ8).

**Figure 12:**
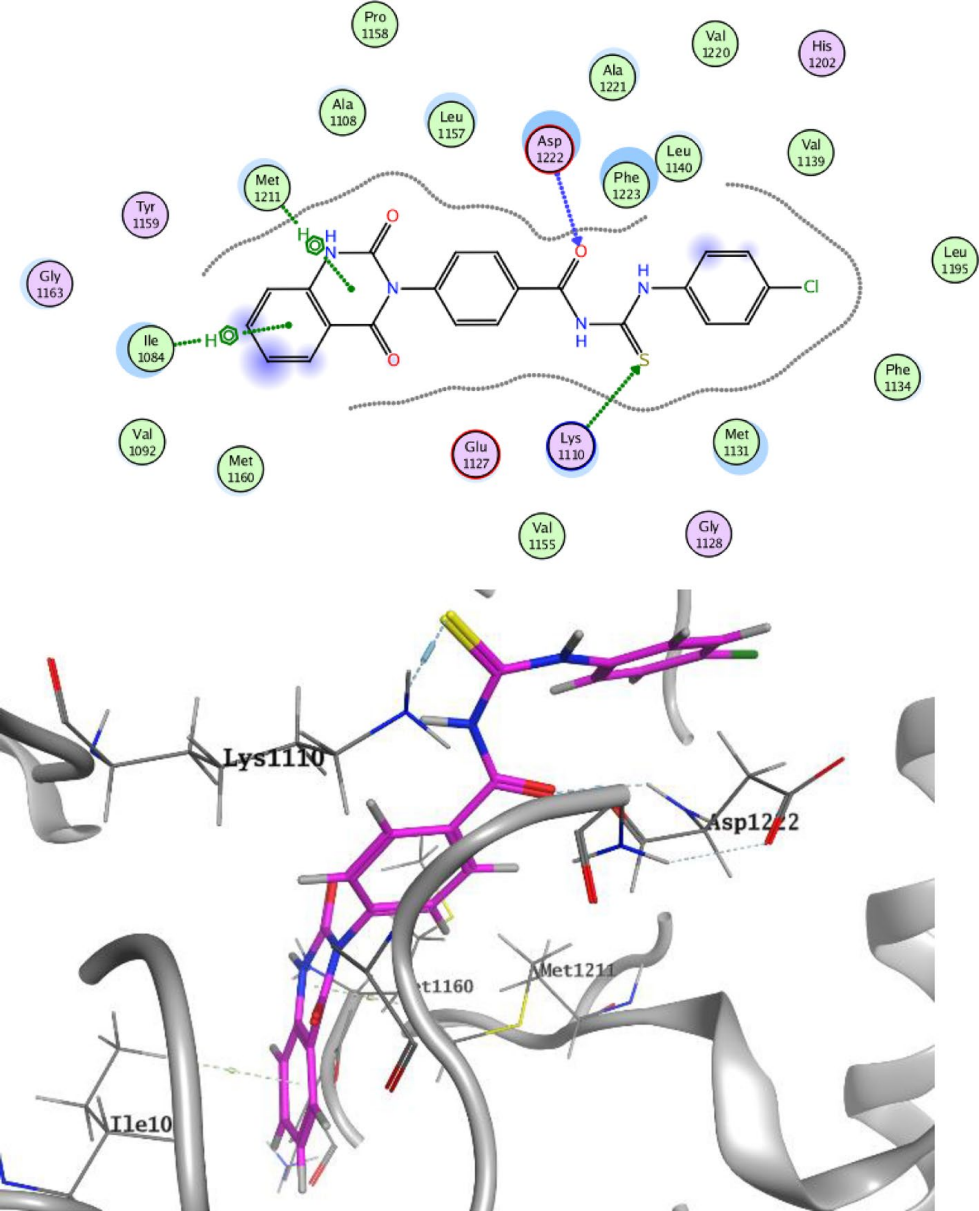
Docking style of compound **3e** with c-Met TK (PDB: 3LQ8).

On the other hand, Figs. 11 and 12 represent docking representations of compounds **3c** and **3e** it the active site of c-Met TK. α-Oxo moiety of quinazoline ring of compound **3c** showed HB with highly conserved residue Met1160 in the hinge region as well as thiourea group formed HB with the another highly conserved residue Lys1110 in the HB region. Further, compound **3c** declared hydrophobic interaction with Ile1084. *N*-acylthiourea group of compound **3e** exhibited dual HB with both Lys 1110 and Asp1222 in addition to hydrophobic interactions with both Ile1084 and Met1211 in the hinge region.

#### In silico prediction of physicochemical and pharmacokinetic properties

To reach advanced clinical phases, drug candidates have to possess an acceptable pharmacokinetic profile. Therefore, the physicochemical and pharmacokinetic properties of target derivatives were predicted using SwissADME (Tables 6, 7 and Fig. 13). All target compounds showed good druglikeness with no Lipinski violations except compound **3h**. Regarding Abbott bioavailability score, most target derivatives have accepted oral absorption. All target compounds as well as cabozantinib were predicted not to cross BBB. Target compounds were predicted to have little effect on CYP450 enzymes like CYP1A2, CYP2C19, CYP2C9, CYP2D6 and CYP3A4 which minimizes the suspected drug-drug interactions.

**Table 6:** Physicochemical parameters and drug-likeness of target compounds and cabozantinib.

#	MW	RB	HBA	HBD	MR	TPSA	iLOGP	Number of violations					Bioavailability score
								Lipinski	Ghose	Veber	Egan	Muegge	
**2a**	365.37	2	4	3	99.20	148.49	1.73	0	0	1	1	0	0.55
**2b**	381.43	2	3	3	103.77	163.51	2.07	0	0	1	1	1	0.55
**3a**	416.45	6	3	3	119.78	128.08	2.99	0	0	0	0	0	0.55
**3b**	460.46	7	5	4	126.74	165.38	2.42	0	0	1	1	1	0.11
**3c**	460.46	7	5	4	126.74	165.38	2.21	0	0	1	1	1	0.11
**3d**	488.52	9	5	3	135.87	154.38	3.26	0	2	1	1	1	0.55
**3e**	450.9	6	3	3	124.79	128.08	3.28	0	0	0	0	0	0.55
**3f**	461.45	7	5	3	128.60	173.9	3.02	0	0	1	1	1	0.55
**3g**	432.45	6	4	4	121.80	148.31	2.7	0	0	1	1	0	0.55
**3h**	573.6	9	7	4	152.48	208.41	2.09	2	2	1	1	1	0.17
**4a**	456.52	6	5	3	120.50	220.9	2.07	0	0	1	1	1	0.55
**4b**	473.53	6	4	3	132.96	169.21	2.7	0	1	1	1	1	0.55
CBZ	501.51	10	7	2	136.59	98.78	3.6	1	2	0	0	1	0.55

CBZ, cabozantinib; RB, rotatable bonds; MR, Molar Refractivity; TPSA, Topological polar surface area.

**Table 7:** Solubility and pharmacokinetics of target compounds and cabozantinib.

#	ESOL Log S	ESOL Class	GI absorption	BBB permeant	Pgp substrate	CYP1A2 inhibitor	CYP2C19 inhibitor	CYP2C9 inhibitor	CYP2D6 inhibitor	CYP3A4 inhibitor
**2a**	− 3.61	Soluble	Low	No	No	No	No	No	No	No
**2b**	− 4.09	Moderately soluble	Low	No	No	No	No	No	No	No
**3a**	− 4.95	Moderately soluble	High	No	No	No	Yes	Yes	No	Yes
**3b**	− 4.81	Moderately soluble	Low	No	No	No	No	Yes	No	No
**3c**	− 4.81	Moderately soluble	Low	No	No	No	No	No	No	No
**3d**	− 5.26	Moderately soluble	Low	No	No	No	Yes	Yes	No	Yes
**3e**	− 5.54	Moderately soluble	High	No	No	No	Yes	Yes	No	Yes
**3f**	− 5.01	Moderately soluble	Low	No	No	No	Yes	Yes	No	Yes
**3g**	− 4.81	Moderately soluble	Low	No	No	No	No	Yes	No	No
**3h**	− 5.16	Moderately soluble	Low	No	No	No	Yes	Yes	No	Yes
**4a**	− 4.71	Moderately soluble	Low	No	No	No	No	No	No	Yes
**4b**	− 5.82	Moderately soluble	Low	No	No	No	Yes	Yes	No	No
CBZ	− 6.13	Poorly soluble	High	No	Yes	No	Yes	Yes	Yes	Yes

**Figure 13:**
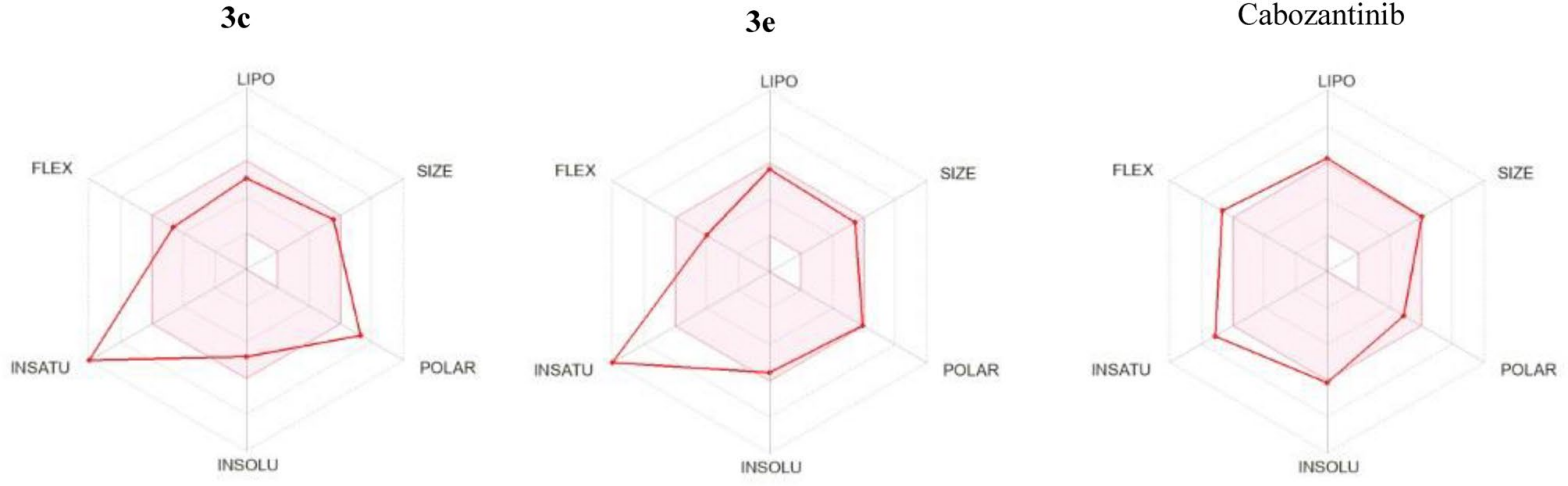
Rader for **3c**, **3e** and cabozantinib*.*

## Conclusion

In brief, based on the structure of a dual VEGFR-2/c-Met inhibitor, cabozantinib, we designed and synthesized a new series of novel 3-phenylquinazolin-2,4(1*H*,3*H*)-diones with (thio)urea scaffold. The cytotoxic activity of the target compounds was conducted against HCT-116 colorectal cancer cell line. Compounds contain electron-withdrawing groups on phenyl ring at position-4, declared the highest cytotoxic activity. The inhibitory activity was performed against both VEGFR-2 and c-Met TKs. α-Oxo moiety in quinazoline ring form HB with Met1160 residue in the adenine region of c-Met TK. Compound **3e** has the highest inhibitory activity against both VEGFR-2/c-Met (IC_50_ = 83 and 48 nM, respectively).

## Experimental

### Chemistry

All reactions were observed on silica gel GF254 plate with thin layer chromatography (TLC). Melting points (uncorrected) were recorded on an electrothermal melting apparatus. FT-IR spectra were recorded on a Shimadzu 8101 PC spectrometer. ^1^H- and ^13^C-NMR spectra were determined on a Varian Mercury 400 MHz spectrophotometer in DMSO-*d*_*6*_. Chemical shifts are reported in parts per million with tetramethylsilane as an internal standard and are given in δ units. Electron impact mass spectra were obtained at 70 eV using a GCMS-QP 1000 EX spectrometer. Elemental analyses were carried out at the microanalytical center at Cairo University.

#### Synthesis of 4-(2,4-dioxo-1,4-dihydro-2*H*-quinazolin-3-yl)-benzoyl chloride 1

4-(2,4-dioxo-1,4-dihydro-2*H*-quinazolin-3-yl)-benzoic acid (0.013 mol, 1 gm) was dissolved in thionyl chloride, then the reaction mixture was refluxed for 5 h. After completion of the reaction, the excess of thionyl chloride was evaporated and the residue was allowed to stand at room temperature, then recrystallized from benzene to yield the product as yellow crystal. Yield: 85%. M.P: 270 °C. FT-IR (KBr, υ, cm^−1^) = 3195 (NH), 1763, 1725 (C = O’s), 751 (C–Cl). ^1^H-NMR (DMSO-*d*_*6*_, 400 MHz): δ (ppm) = 11.65 (s, 1H, NH), 7.22–7.96 (m, 4H, Ar–H (quinazoline)), 7.49–8.07 (dd, 4H, Ar–H). ^13^C-NMR (DMSO): δ 114.74, 115.77, 123.09, 127.52, 128.04, 129.99, 130.30, 131.95, 135.81, 140.30, 150.43, 162.57, 167.35. MS (EI) (RT: 3.56–3.58 min): *m/z* = 300.22 (obs.), 300.03 (expected) [M^+^], 302.22 (obs.), 302.27 (expected) [M^+^ + 2]. *Anal. Calcd* for C_15_H_9_ClN_2_O_3_: C, 59.91; H, 3.02; Cl, 11.79; N, 9.32%. Found C, 60.16; H, 3.26; Cl, 11.84; N, 9.18%.

#### General procedures for the synthesis of 3-[4-(6-mercapto-4-oxo/thioxo-1,4-dihydro-[1,3,5]-triazin-2-yl)-phenyl]-1*H*-quinazolin-2,4-dione 2a,b

4-(2,4-dioxo-1,4-dihydro-2*H*-quinazolin-3-yl)-benzoyl chloride **1** (0.003 mol, 0.32 g) was allowed to react with ammonium thiocyanate (0.003 mol, 0.32 g) in dioxane (20 ml) under reflux for 2 h. After completion of the reaction, the formed precipitate of ammonium chloride was removed, then urea and/or thiourea was added to the filtrate in presence of few drops of TEA under reflux for 6 h. The solid formed was filtered off, dried, and recrystallized from ethanol to afford compounds **2a, b**, respectively.

##### 3-(4-(4-oxo-6-thioxo-1,4,5,6-tetrahydro-1,3,5-triazin-2-yl)phenyl)quinazolin-2,4(1H,3H)-dione 2a

Brown crystals. Yield 60%. MP > 300 °C. FT-IR (KBr, υ, cm^−1^) = 3406, 3226, 3060 (NH’s), 1717, 1654 (C = O’s), 1610 (C=N), 1280 (C=S). ^1^H-NMR (DMSO-*d*_*6*_, 400 MHz): δ (ppm) = 11.64 (s, 1H, NH), 8.10 (s, 1H, NH), 7.98 (s, 1H, NH), 7.25–7.75 (m, 4H, Ar–H (quinazoline), 7.42–7.96 (dd, 4H, Ar–H). ^13^C-NMR (DMSO): δ 114.76, 115.75, 123.06, 128.05, 128.47, 129.52, 129.71, 129.78, 134.63, 135.78, 138.78, 140.31, 150.50, 162.60, 167.94. MS (EI) (RT: 2.90–3.00): *m/z* = 365.00 (obs.), 365.37 (expected) [M^+^], 367.00 (obs.), 367.37 (expected) [M^+^ + 2]. *Anal. Calcd* for C_17_H_11_N_5_O_3_S: C, 55.88; H, 3.03; N, 19.17; S, 8.78%. Found C, 55.99; H, 3.14; N, 19.10; S, 8.88%.

##### 3-(4-(4,6-dithioxo-1,4,5,6-tetrahydro-1,3,5-triazin-2-yl)phenyl)quinazolin-2,4(1H,3H)-dione 2b

Pale brown crystals. Yield 60%. MP > 300 °C. FT-IR (KBr, υ, cm^−1^) = 3406, 3235, 3063 (NH's), 1716, 1655 (C=O’s), 1607 (C=N), 1275, 1237 (C=S’s). ^1^H-NMR (DMSO-*d*_*6*_, 400 MHz): δ (ppm) = 11.64 (s, 1H, NH), 8.08 (s, 1H, NH), 8.06 (s, 1H, NH), 7.23–8.04 (m, 4H, Ar–H (quinazoline)), 7.42–7.98 (dd, 4H, Ar–H). ^13^C-NMR (DMSO): δ 114.76, 115.75, 123.06, 128.05, 128.48, 129.72, 132.45, 134.64, 135.68, 138.48, 140.30, 150.22, 162.17, 167.96, 182.55. MS (EI) (RT: 1.60–1.63): *m/z* = 381.00 (obs.), 381.44 (expected) [M^+^], 383.00 (obs.), 383.44 (expected) [M^+^ + 2]. *Anal. Calcd* for C_17_H_11_N_5_O_2_S_2_: C, 53.53; H, 2.91; N, 18.36; S, 16.81%. Found C, 53.66; H, 3.09; N, 18.25; S, 16.97%.

#### General procedures for the synthesis of arylquinazolin-2,4-diones 3a-h

Multi-component reaction of 4-(2,4-dioxo-1,4-dihydro-2*H*-quinazolin-3-yl)-benzoyl chloride **1** with ammonium thiocyanate and aromatic amines namely aniline, *m*-aminobenzoic acid, *p*-aminobenzoic acid, ethyl 4-aminobenzoate, *p*-chloroaniline, *p*-nitroaniline,* p*-aminophenol, and 4-amino-*N*-pyrimidin-2-yl-benzenesulfonamide in dioxane (20 ml) and few drops of TEA was heated under reflux for 10–12 h (monitored by TLC) to afforded compounds **3a–h**, respectively.

##### 4-(2,4-dioxo-1,4-dihydroquinazolin-3(2H)-yl)-N-(phenylcarbamothioyl)benzamide 3a

White crystals. Yield 75%. MP 210 °C. FT-IR (KBr, υ, cm^−1^) = 3243 (NH), 1716, 1663 (C=O’s), 1268 (C=S). ^1^H-NMR (DMSO-*d*_*6*_, 400 MHz): δ (ppm) = 12.63 (s, 1H, NH), 11.72 (s, 1H, NH), 11.66 (s, 1H, NH), 7.26–8.05 (m, 9H, Ar–H), 7.72–8.11 (dd, 4H, Ar–H). ^13^C-NMR (DMSO): δ 114.78, 115.79, 123.10,124.89, 126.85, 128.06, 128.81, 129.18 129.71, 129.80, 132.45, 135.84, 140.33, 140.48, 150.43, 162.59, 168.27, 179.58. MS (EI) (RT: 4.47–4.50): *m/z* = 416.34 (obs.), 416.34.46 (expected) [M^+^]. *Anal. Calcd* for C_22_H_16_N_4_O_3_S: C, 63.45; H, 3.87; N, 13.45; S, 7.70%. Found C, 63.56; H, 4.01; N, 13.35; S, 7.83%.

##### 3-(3-(4-(2,4-dioxo-1,4-dihydroquinazolin-3(2H)-yl)benzoyl)thioureido)benzoic acid 3b

Pale brown crystals. Yield 75%. MP: > 300 °C. FT-IR (KBr, υ, cm^−1^) = 3405 (COOH), 3222, 3061 (NH's), 1716, 1656 (C=O’s), 1279 (C=S). ^1^H-NMR (DMSO-*d*_*6*_, 400 MHz): δ (ppm) = 12.70 (s, 1H, COOH), 11.65 (s, 1H, NH), 11.61 (s, 1H, NH), 11.40 (s, 1H, NH), 7.23–8.12 (m, 8H, Ar–H), 7.42–7.98 (dd, 4H, Ar–H). ^13^C-NMR (DMSO): δ 114.75, 115.75, 123.06, 123.15, 128.05, 128.48, 129.52, 129.72, 129.78, 129.86, 134.63, 135.77, 138.79, 140.30, 150.51, 162.61, 167.97, 179.94, 182.54. MS (EI) (RT: 2.73–2.76): *m/z* = 460.48 (obs.), 460.47 (expected) [M^+^]. *Anal. Calcd* for C_23_H_16_N_4_O_5_S: C, 59.99; H, 3.50; N, 12.17; S, 6.96%. Found C, 60.12; H, 3.60; N, 12.09; S, 7.11%.

##### 4-(3-(4-(2,4-dioxo-1,4-dihydroquinazolin-3(2H)-yl)benzoyl)thioureido)benzoic acid 3c

Orange crystal crystals. Yield 70%. MP 258 °C. FT-IR (KBr, υ, cm^−1^) = 3355 (COOH), 3044 (NH), 1721, 1681 (C=O’s), 1264 (C=S). ^1^H-NMR (DMSO-*d*_*6*_, 400 MHz): δ (ppm) = 12.80 (s, 1H, COOH), 12.68 (s, 1H, NH), 11.82 (s, 1H, NH), 11.65 (s, 1H, NH), 7.25–8.16 (m, 8H, Ar–H), 7.52–8.02 (dd, 4H, Ar–H). ^13^C-NMR (DMSO): δ 113.01, 114.77, 115.79, 117.30, 123.10, 124.04, 128.05, 128.57, 129.85, 130.42, 131.69, 132.39, 140.54, 142.43, 150.43, 162.59, 167.20, 168.18, 179.74. MS (EI) (RT: 4.09–4.40): *m/z* = 460.48 (obs.), 460.47(expected) [M^+^]. *Anal. Calcd* for C_23_H_16_N_4_O_5_S: C, 59.99; H, 3.50; N, 12.17; S, 6.96%. Found C, 60.12; H, 3.60; N, 12.09; S, 7.11%.

##### Ethyl4-(3-(4-(2,4-dioxo-1,4-dihydroquinazolin-3(2H)-yl)benzoyl)thioureido)benzoate 3d

White crystals. Yield 80%. MP > 300 °C. FT-IR (KBr, υ, cm^−1^) = 3359, 3065, 2932 (NH's), 1715, 1666 (C=O’s), 1270 (C=S). ^1^H-NMR (DMSO-*d*_*6*_, 400 MHz): δ (ppm) = 11.65 (s, 1H, NH), 11.45 (s, 1H, NH) 10.72 (s, 1H, NH), 7.24–8.00 (m, 8H, Ar–H), 7.54–8.08 (dd, 4H, Ar–H), 4.35 (q, 2H, CH_2_), 1.36 (t, 3H, CH_3_). ^13^C-NMR (DMSO): δ 14.72, 60.98, 114.78, 115.78, 120.02, 123.08, 125.11, 128.06 128.87, 129.80, 130.59, 134.96, 135.81, 139.36, 140.34, 144.07, 150.49, 162.62, 165.25, 165.84, 166.16. MS (EI) (RT: 1.91–1.95): *m/z* = 488.05 (obs.), 488.53 (expected) [M^+^]. *Anal. Calcd* for C_25_H_20_N_4_O_5_S: C, 61.47; H, 4.13; N, 11.47; S, 6.56%. Found C, 61.56; H, 4.29; N, 11.35; S, 6.66%.

##### N-((4-chlorophenyl)carbamothioyl)-4-(2,4-dioxo-1,4-dihydroquinazolin-3(2H)-yl) benzamide 3e

Pale violet crystals. Yield 81%. MP 250 °C. FT-IR (KBr, υ, cm^−1^) = 3323, 3248 (NH's), 1724, 1671 (C=O’s), 1245 (C=S). ^1^H-NMR (DMSO-*d*_*6*_, 400 MHz): δ (ppm) = 11.67 (s, 1H, NH), 10.60 (s, 1H, NH), 10.36 (s, 1H, NH), 7.29–8.11 (m, 8H, Ar–H), 7.43–7.97 (dd, 4H, Ar–H). ^13^C-NMR (DMSO): δ 114.74, 115.79, 123.05, 128.02, 128.48, 129.02, 129.41, 129.50, 129.74, 132.17, 134.61, 135.76, 138.78, 140.33, 150.47, 162.60, 167.47, 167.93. MS (EI) (RT: 1.74–1.76): *m/z* = 450.33 (obs.), 450.91 (expected) [M^+^], 452.33 (obs.), 452.91 (expected) [M^+^ + 2]. *Anal. Calcd* for C_22_H_15_ClN_4_O_3_S: C, 58.60; H, 3.35; Cl, 7.86; N, 12.43; S, 7.11%. Found C, 58.74; H, 3.46; Cl, 7.91; N, 12.38; S, 7.22%.

##### 4-(2,4-dioxo-1,4-dihydroquinazolin-3(2H)-yl)-N-((4-nitrophenyl)carbamothioyl) benzamide 3f.

Brown crystals. Yield 65%. MP 255 °C. FT-IR (KBr, υ, cm^−1^) = 3393, 3247, 3071 (NH's), 1718, 1667 (C = O's), 1237 (C = S). ^1^H-NMR (DMSO-*d*_*6*_, 400 MHz): δ (ppm) = 11.90 (s, 1H, NH), 11.64 (s, 1H, NH), 11.40 (s, 1H, NH), 7.24–7.97 (m, 8H, Ar–H), 7.50–8.06 (dd, 4H, Ar–H). ^13^C-NMR (DMSO): δ 114.77, 115.79, 120.61, 122.10, 123.08, 125.06, 128.05, 129.69, 129.78, 132.55, 135.80, 135.82, 139.75, 140.34, 150.41, 162.57, 167.75, 182.55. MS (EI) (RT: 5.60–5.85): *m/z* = 459.00 (obs.), 459.46 (expected) [M^+^-2], 461.00 (obs.), 461.46 (expected) [M^+^]. *Anal. Calcd* for C_22_H_15_N_5_O_5_S: C, 57.26; H, 3.28; N, 15.18; S, 6.95%. Found C, 57.40; H, 3.40; N, 15.09; S, 7.08%.

##### 4-(2,4-dioxo-1,4-dihydroquinazolin-3(2H)-yl)-N-((4-hydroxyphenyl)carbamothioyl) benzamide 3g

Dark brown crystals. Yield 75%. MP 260 °C. FT-IR (KBr, υ, cm^-1^) = 3386 (OH), 3246, 3067, 2919 (NH's), 1716, 1665 (C=O's), 1268 (C=S). ^1^H-NMR (DMSO-*d*_*6*_, 400 MHz): δ (ppm) = 12.44 (s, 1H, OH), 11.64 (s, 1H, NH), 11.62 (s, 1H, NH), 11.40 (s, 1H, NH), 6.81–8.10 (m, 8H, Ar–H), 7.50–8.06 (dd, 4H, Ar–H). ^13^C-NMR (DMSO): δ 114.78, 115.57, 115.78, 120.04, 123.09, 126.47, 128.05, 128.48, 129.70, 129.78, 132.55, 135.82, 140.33, 150.42, 162.57, 167.76, 168.10, 182.54. MS (EI) (RT: 3.40–3.75): *m/z* = 432.00 (obs.), 432.45 (expected) [M^+^]. *Anal. Calcd* for C_22_H_16_N_4_O_4_S: C, 61.10; H, 3.73; N, 12.96; S, 7.41%. Found C, 61.22; H, 3.85; N, 12.85; S, 7.55%.

#### 4-(2,4-dioxo-1,4-dihydroquinazolin-3(2H)-yl)-N-((4-(N-(pyrimidin-2-yl)sulfamoyl) phenyl)carbamothioyl)benzamide 3h

Pale brown crystals. Yield 80%. MP 300 ºC. FT-IR (KBr, υ, cm^−1^) = 3397, 3094, 2920 (NH's), 1718, 1666 (C=O’s), 15,835 (C=N), 1337 (O=S=O), 1263 (C=S). ^1^H-NMR (DMSO-*d*_*6*_, 400 MHz): δ (ppm) = 12.76 (S, 1H, NH), 11.83 (s, 1H, NH), 11.63 (s, 1H, NH), 11.40 (s, 1H, NH), 7.08–8.55 (m, 11H, Ar–H + pyrimidine protons), 7.50–8.05 (dd, 4H, Ar–H). ^13^C-NMR (CDCl_3_): δ 114.77, 115.78, 122.10, 123.09, 123.85, 124.35, 127.57, 128.05, 128.75, 129.71, 129.78, 129.86, 132.55, 135.82, 140.32, 140.35, 142.75, 150.42, 162.57, 167.76, 182.54. MS (EI) (RT: 2.50–2.75): *m/z* = 573.00 (obs.), 573.54 (expected) [M^+^], 575.00 (obs.), 575.54 (expected) [M^+^ + 2]. *Anal.* Calcd for C_26_H_19_N_7_O_5_S_2_: C, 54.44; H, 3.34; N, 17.09; S, 11.18%. Found C, 54.66; H, 3.64; N, 17.00; S, 11.33%.

#### General procedures for the synthesis of heterylquinazolin-2,4-diones 4a-b

A mixture of 4-(2,4-dioxo-1,4-dihydro-2*H*-quinazolin-3-yl)-benzoyl chloride **1** (0.005 mol, 1.5 gm) and ammonium thiocyanate (0.005 mol, 0.39 gm) in dioxane (20 ml) and few drops of TEA was refluxed for 2 h. Afterward, the formed ammonium chloride precipitate was removed by filtration, then hetero amines namely 5-amino-[1,3,4]-thiadiazole-2-thiol, and 2-aminobenzothiazole (0.005 mol) were added to the filtrate and the reaction mixture was refluxed for 8–10 h. The formed solids were filtered off, purified by recrystallization using benzene/ethanol to furnish compounds **4a-b**, respectively.

##### 4-(2,4-dioxo-1,4-dihydroquinazolin-3(2H)-yl)-N-((5-mercapto-1,3,4-thiadiazol-2-yl)carbamothioyl)benzamide 4a

Yellow crystals. Yield 82%. MP > 300 °C. FT-IR (KBr, υ, cm^−1^) = 3408, 3212, 3064 (NH's), 2723 (SH), 1717, 1655 (C=O’s), 1608 (C=N), 1275 (C=S). ^1^H-NMR (DMSO-*d*_*6*_, 400 MHz): δ (ppm) = 13.16 (s, 1H, SH), 11.68 (s, 1H, NH), 11.63 (s, 1H, NH), 11.61 (s, 1H, NH), 7.24–8.08 (m, 4H, Ar–H(quinazoline)), 7.42–7.98 (dd, 4H, Ar–H). ^13^C-NMR (DMSO): δ 114.76, 115.75, 123.06, 128.05, 128.47, 129.52, 130.28, 131.04, 134.64, 135.77, 138.78, 140.30, 150.43, 162.60, 167.35, 167.94. MS (EI) (RT: 3.30–3.50): *m/z* = 455.00 (obs.), 455.53 (expected) [M^+^-1], 456.00 (obs.), 456.53 (expected) [M^+^]. *Anal. Calcd* for C_18_H_12_N_6_O_3_S_3_: C, 47.36; H, 2.65; N, 18.41; S, 21.07%. Found C, 47.50; H, 2.78; N, 18.32; S, 21.21%.

##### N-(benzo[d]thiazol-2-ylcarbamothioyl)-4-(2,4-dioxo-1,4-dihydroquinazolin-3(2H)-yl)benzamide 4b

Brown crystals. Yield 75%. MP > 300 °C. FT-IR (KBr, υ, cm^−1^) = 3406, 3234, 3063 (NH's), 1716, 1655 (C=O’s), 1608 (C=N), 1280 (C=S). ^1^H-NMR (DMSO-*d*_*6*_, 400 MHz): δ (ppm) = 11.57 (s, 2H, 2NH), 11.40 (s, 1H, NH), 7.23–8.08 (m, 8H, Ar–H), 7.42–7.98 (dd, 4H, Ar–H). ^13^C-NMR (CDCl_3_): δ 114.76, 115.75, 123.06, 124.05, 128.05, 128.46, 129.52, 129.71, 129.78, 132.51, 134.63, 135.08, 135.78, 138.78, 140.30, 146.19, 150.42, 150.50, 162.60, 167.76, 167.93. MS (EI) (RT: 1.90–2.10): *m/z* = 473.00 (obs.), 473.54 (expected) [M^+^], 474.00 (obs.), 474.54 (expected) [M^+^ + 1]. *Anal. Calcd* for C_23_H_15_N_5_O_3_S_2_: C, 58.34; H, 3.19; N, 14.79; S, 13.54%. Found C, 58.50; H, 3.29; N, 14.62; S, 13.70%.

### Biological evaluation

Supporting information includes all experimental details for the MTT assay, and in vitro enzyme inhibition of VEGFR-2 and c-Met TKs.

### In silico studies

#### Molecular modeling study

Molecular docking and visualizations of 2D and 3D styles were conducted by using MOE 2014.0901 and Discovery Studio visualizer softwares. The structures of co-crystallized TKs were retrieved from protein data bank (VEGFR-2: 4ASD & c-Met: 3LQ8). The structures of compound **3c** and **3e** were standardized and their energies were minimized. After that, the protein structures were prepared using MOE standard protocol^27^. Validation of docking approach was performed by redocking process of the original ligands (VEGFR-2: sorafenib & c-Met: foretinib) into the binding sites of both proteins^9^. Docking of compounds **3c** and **3e** were performed using default MOE docking setting. The 2D and 3D representations of the most stable poses were selected for further studies.

#### In silico prediction of physicochemical and pharmacokinetic properties

The freely available SwissADME website was utilized to predict the physicochemical and pharmacokinetics of the target compounds. Supporting information includes how different filters and parameters were calculated^9^.

### Supplementary Information

Supplementary Information.

## Data Availability

All data generated or analysed during this study are included in the supplementary information file.
