# Supplementary material for: Novel 3-phenylquinazolin-2,4(1H,3H)-diones as dual VEGFR-2/c-Met-TK inhibitors: design, synthesis, and biological evaluation
Source: Sci Rep. 2023 Oct 30;13:18567. doi: 10.1038/s41598-023-45687-y (PMC10616113; doi:10.1038/s41598-023-45687-y)
Supplement: Supplementary file 1 — Supplementary Information. [file 41598_2023_45687_MOESM1_ESM.docx]

**Novel 3-****phenylquinazolin-2,4(1*H*,3*H*)-diones as dual VEGFR-2/c-Met-TK inhibitors: Design, synthesis, and biological evaluation**

Abdelfattah Hassan^1*^, Ahmed M. Mosallam^2^, Amal O. A. Ibrahim^2^, Mohamed Badr^3^, Aboubakr H. Abdelmonsef^2*^

*^1^Department of Medicinal Chemistry, Faculty of Pharmacy, South Valley University, Qena, Egypt*

^2^*Department of Chemistry, Faculty of Science, South Valley University, Qena, Egypt*

^3^*Department of Biochemistry, Faculty of Pharmacy, Menoufia University, Menoufia, Egypt*

*To whom correspondence should be addressed:*

^*^Abdelfattah Hassan: [abdelfattah_hassan@svu.edu.eg](mailto:abdelfattah_hassan@svu.edu.eg), Tel.: +201000760083

^*^Aboubakr H. Abdelmonsef: aboubakr.ahmed@sci.svu.edu.eg; Tel.: +201098965494

**List of Figures**

[**Figure S1**: IR spectrum of compound **2a**. 1](#_Toc140782287)

[**Figure S2**: ^1^H-NMR spectrum of compound **2a**. 2](#_Toc140782288)

[**Figure S3**: ^13^C-NMR spectrum of compound **2a**. 3](#_Toc140782289)

[**Figure S4**: Mass spectrum of compound **2a**. 4](#_Toc140782290)

[**Figure S5**: IR spectrum of compound **2b**. 5](#_Toc140782291)

[**Figure S6**: ^1^H-NMR spectrum of compound **2b**. 6](#_Toc140782292)

[**Figure S7**: ^13^C-NMR spectrum of compound **2b**. 7](#_Toc140782293)

[**Figure S8**: Mass spectrum of compound **2b**. 8](#_Toc140782294)

[**Figure S9**: IR spectrum of compound **3a**. 9](#_Toc140782295)

[**Figure S10**: ^1^H-NMR spectrum of compound **3a**. 10](#_Toc140782296)

[**Figure S11**: ^13^C-NMR spectrum of compound **3a**. 11](#_Toc140782297)

[**Figure S12**: Mass spectrum of compound **3a**. 12](#_Toc140782298)

[**Figure S13**: IR spectrum of compound **3b**. 13](#_Toc140782299)

[**Figure S14**: ^1^H-NMR spectrum of compound **3b**. 14](#_Toc140782300)

[**Figure S15**: ^13^C-NMR spectrum of compound **3b**. 15](#_Toc140782301)

[**Figure S16**: Mass spectrum of compound **3b**. 16](#_Toc140782302)

[**Figure S17**: IR spectrum of compound **3c**. 17](#_Toc140782303)

[**Figure S18**: ^1^H-NMR spectrum of compound **3c**. 18](#_Toc140782304)

[**Figure S19**: ^13^C-NMR spectrum of compound **3c**. 19](#_Toc140782305)

[**Figure S20**: Mass spectrum of compound **3c**. 20](#_Toc140782306)

[**Figure S21**: IR spectrum of compound **3d**. 21](#_Toc140782307)

[**Figure S22**: ^1^H-NMR spectrum of compound 3d. 22](#_Toc140782308)

[**Figure S23**: ^13^C-NMR spectrum of compound **3d**. 23](#_Toc140782309)

[**Figure S24**: Mass spectrum of compound **3d**. 24](#_Toc140782310)

[**Figure S25**: IR spectrum of compound **3e**. 25](#_Toc140782311)

[**Figure S26**: ^1^H-NMR spectrum of compound **3e**. 26](#_Toc140782312)

[**Figure S27**: ^13^C-NMR spectrum of compound **3e**. 27](#_Toc140782313)

[**Figure S28**: Mass spectrum of compound **3e**. 28](#_Toc140782314)

[**Figure S29**: IR spectrum of compound **3f**. 29](#_Toc140782315)

[**Figure S30**: ^1^H-NMR spectrum of compound **3f**. 30](#_Toc140782316)

[**Figure S31**: ^13^C-NMR spectrum of compound **3f**. 31](#_Toc140782317)

[**Figure S32**: Mass spectrum of compound **3f**. 32](#_Toc140782318)

[**Figure S33**: IR spectrum of compound **3g**. 33](#_Toc140782319)

[**Figure S34**: ^1^H-NMR spectrum of compound **3g**. 34](#_Toc140782320)

[**Figure S35**: ^13^C-NMR spectrum of compound **3g**. 35](#_Toc140782321)

[**Figure S36**: Mass spectrum of compound **3g**. 36](#_Toc140782322)

[**Figure S37**: IR spectrum of compound **3h**. 37](#_Toc140782323)

[**Figure S38**: ^1^H-NMR spectrum of compound **3h**. 38](#_Toc140782324)

[**Figure S39**: ^13^C-NMR spectrum of compound **3h**. 39](#_Toc140782325)

[**Figure S40**: Mass spectrum of compound **3h**. 40](#_Toc140782326)

[**Figure S41**: IR spectrum of compound **4a**. 41](#_Toc140782327)

[**Figure S42**: ^1^H-NMR spectrum of compound **4a**. 42](#_Toc140782328)

[**Figure S43**: ^13^C-NMR spectrum of compound **4a**. 43](#_Toc140782329)

[**Figure S44**: Mass spectrum of compound **4a**. 44](#_Toc140782330)

[**Figure S45**: IR spectrum of compound **4b**. 45](#_Toc140782331)

[**Figure S46**: ^1^H-NMR spectrum of compound **4b**. 46](#_Toc140782332)

[**Figure S47**: ^13^C-NMR spectrum of compound **4b**. 47](#_Toc140782333)

[**Figure S48**: Mass spectrum of compound **4b**. 48](#_Toc140782334)

[**Figure S49**: Docking style of compound **2a** with VEGFR-2 TK (PDB: 4asd). 49](#_Toc140782335)

[**Figure S50**: Docking style of compound **2b** with VEGFR-2 TK (PDB: 4asd). 50](#_Toc140782336)

[**Figure S51**: Docking style of compound **3b** with VEGFR-2 TK (PDB: 4asd). 50](#_Toc140782337)

[**Figure S52**: Docking style of compound **3c** with VEGFR-2 TK (PDB: 4asd). 51](#_Toc140782338)

[**Figure S53**: Docking style of compound **3d** with VEGFR-2 TK (PDB: 4asd). 51](#_Toc140782339)

[**Figure S54**: Docking style of compound **3e** with VEGFR-2 TK (PDB: 4asd). 52](#_Toc140782340)

[**Figure S55**: Docking style of compound **3f** with VEGFR-2 TK (PDB: 4asd). 52](#_Toc140782341)

[**Figure S56**: Docking style of compound **3g** with VEGFR-2 TK (PDB: 4asd). 53](#_Toc140782342)

[**Figure S57**: Docking style of compound **3h** with VEGFR-2 TK (PDB: 4asd). 53](#_Toc140782343)

[**Figure S58**: Docking style of compound **4a** with VEGFR-2 TK (PDB: 4asd). 54](#_Toc140782344)

[**Figure S59**: Docking style of compound **4b** with VEGFR-2 TK (PDB: 4asd). 54](#_Toc140782345)

[**Figure S60**: Docking style of compound **3b** with c-Met TK (PDB: 3lq8). 55](#_Toc140782346)

[**Figure S61**: Docking style of compound **3c** with c-Met TK (PDB: 3lq8). 55](#_Toc140782347)

[**Figure S62**: Docking style of compound **3d** with c-Met TK (PDB: 3lq8). 56](#_Toc140782348)

[**Figure S63**: Docking style of compound **3e** with c-Met TK (PDB: 3lq8). 56](#_Toc140782349)

[**Figure S64**: Docking style of compound **3f** with c-Met TK (PDB: 3lq8). 57](#_Toc140782350)

[**Figure S65**: Docking style of compound **3g** with c-Met TK (PDB: 3lq8). 58](#_Toc140782351)

[**Figure S66**: Docking style of compound **3h** with c-Met TK (PDB: 3lq8). 58](#_Toc140782352)

[**Figure S67**: Docking style of compound **4a** with c-Met TK (PDB: 3lq8). 59](#_Toc140782353)

[**Figure S68**: Docking style of compound **4b** with c-Met TK (PDB: 3lq8). 59](#_Toc140782354)


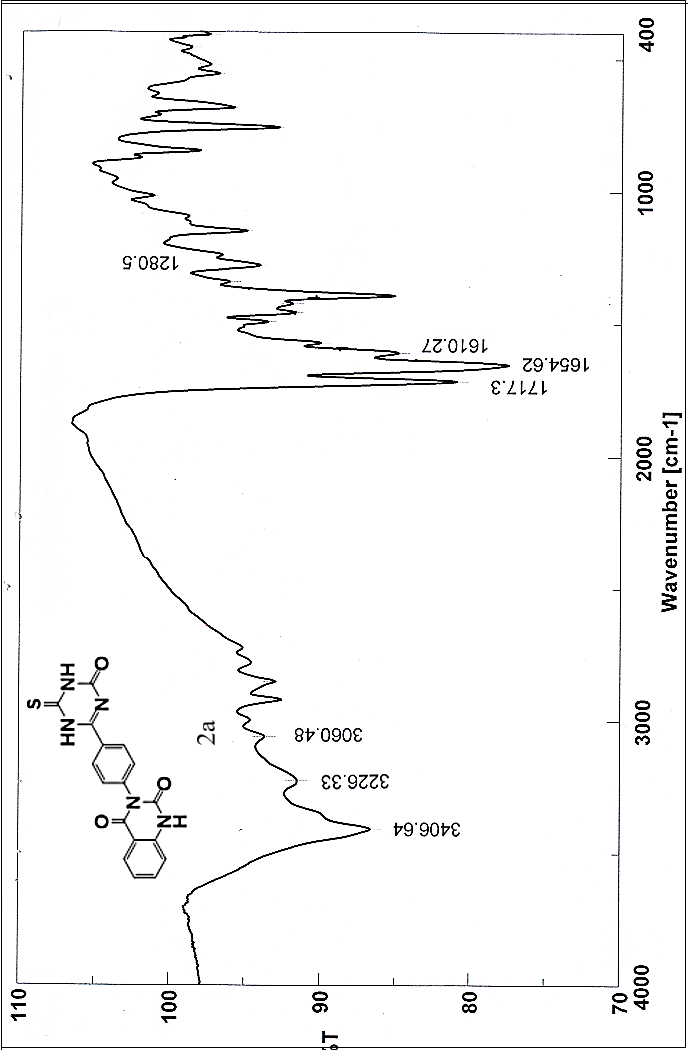


**Figure S1**: IR spectrum of compound **2a**.


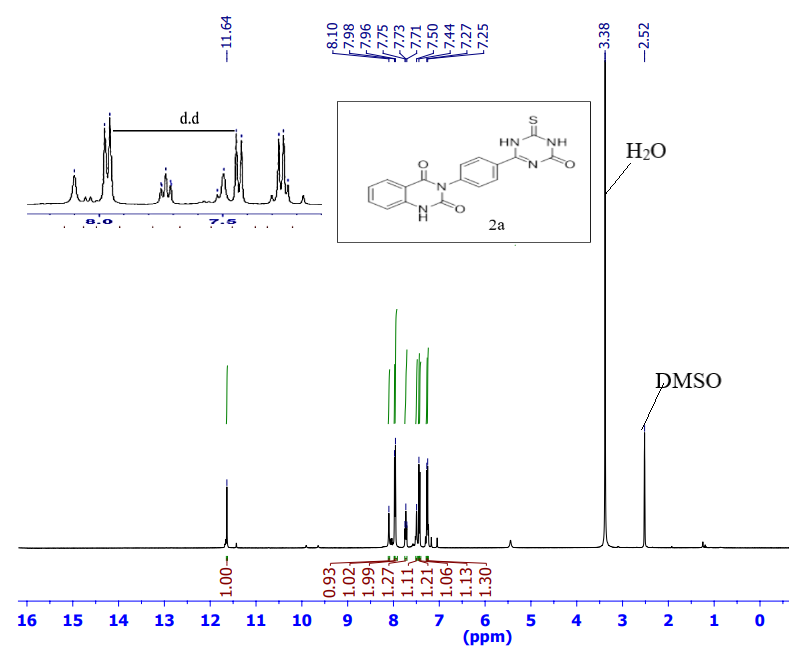


**Figure S2**: ^1^H-NMR spectrum of compound **2a**.


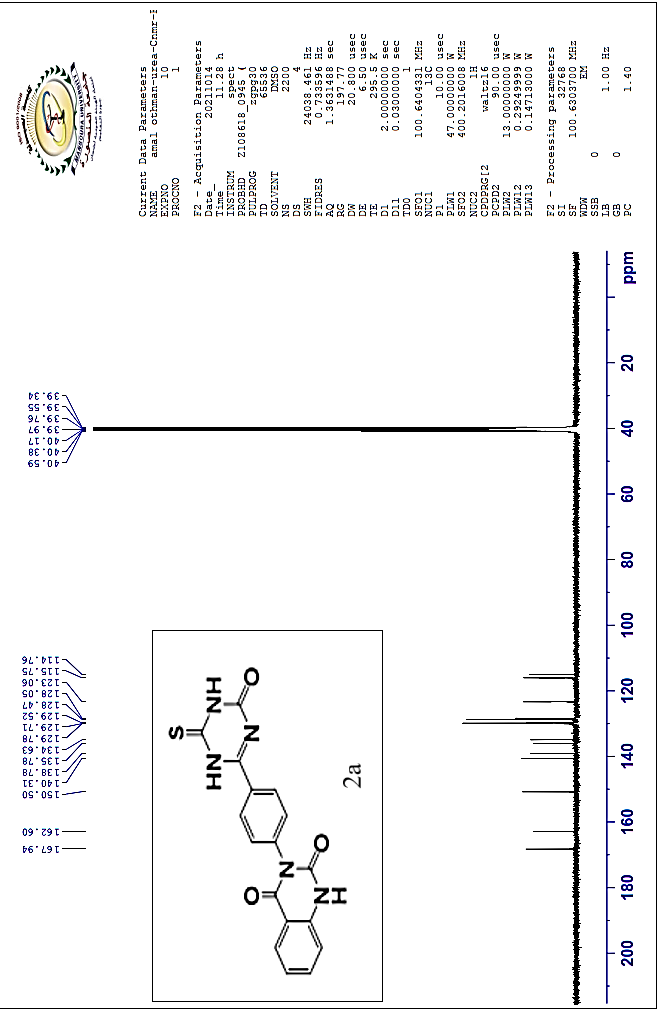


**Figure S3**: ^13^C-NMR spectrum of compound **2a**.


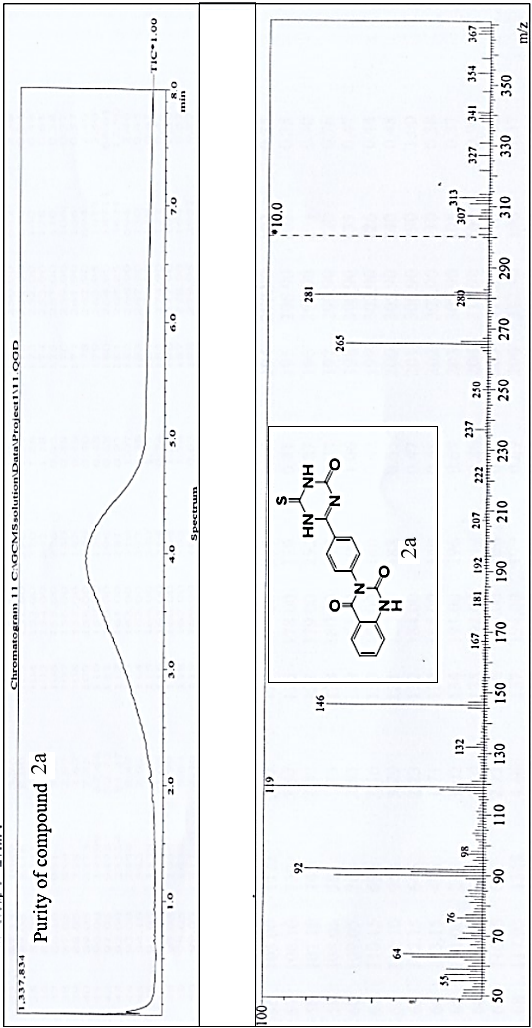


**Figure S4**: Mass spectrum of compound **2a**.


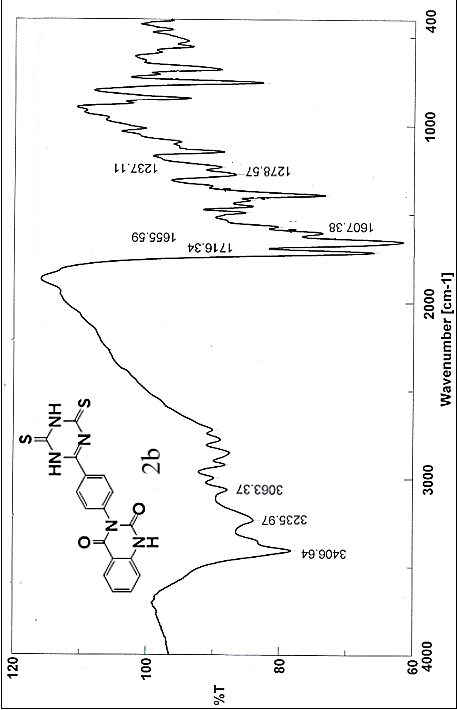


**Figure S5**: IR spectrum of compound **2b**.


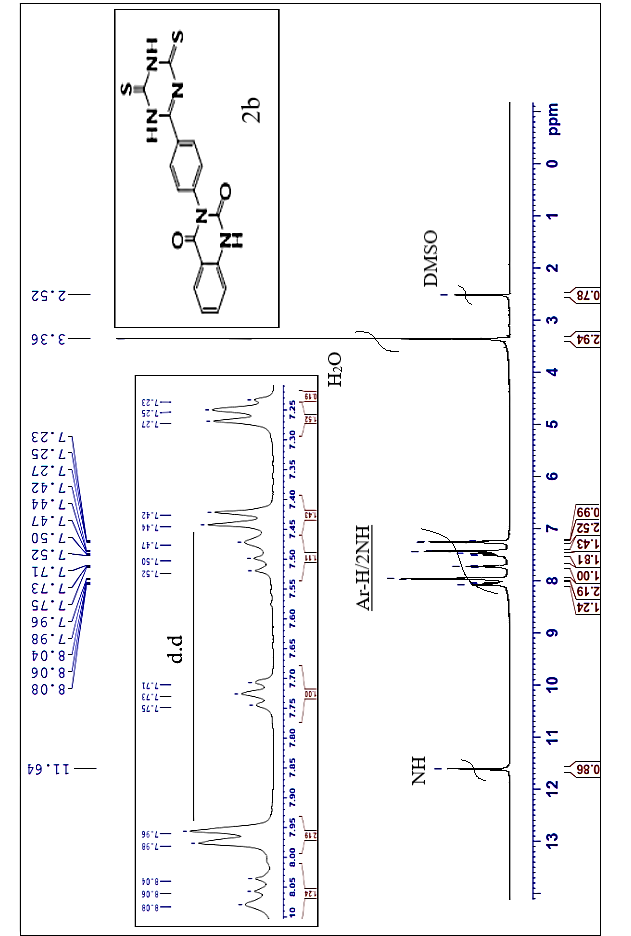


**Figure S6**: ^1^H-NMR spectrum of compound **2b**.


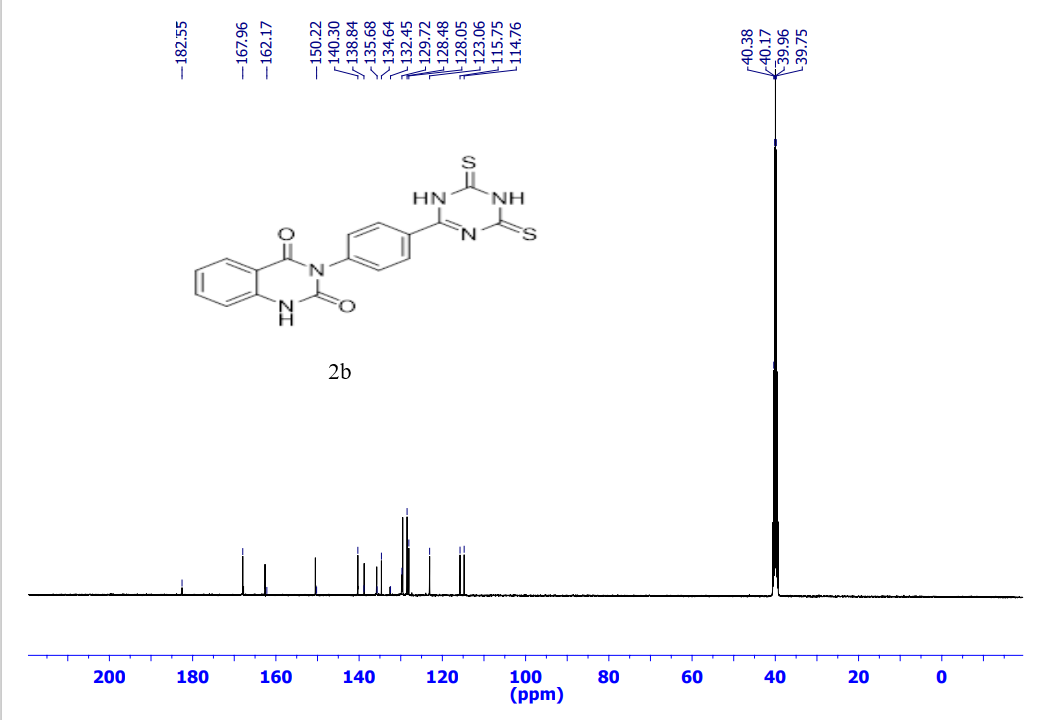


**Figure S7**: ^13^C-NMR spectrum of compound **2b**.


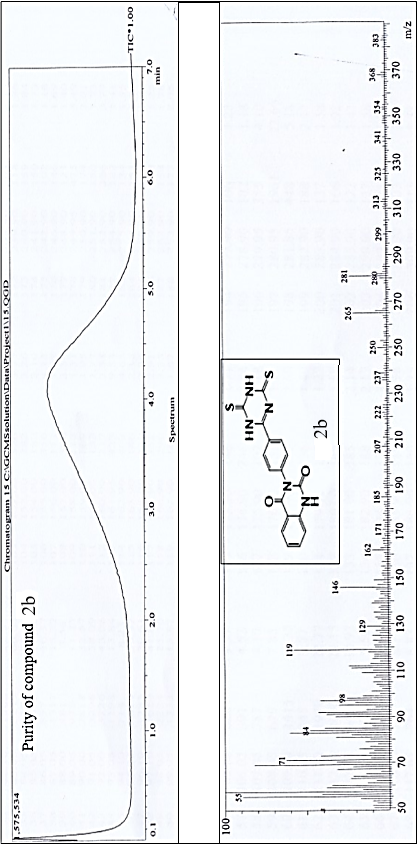


**Figure S8**: Mass spectrum of compound **2b**.


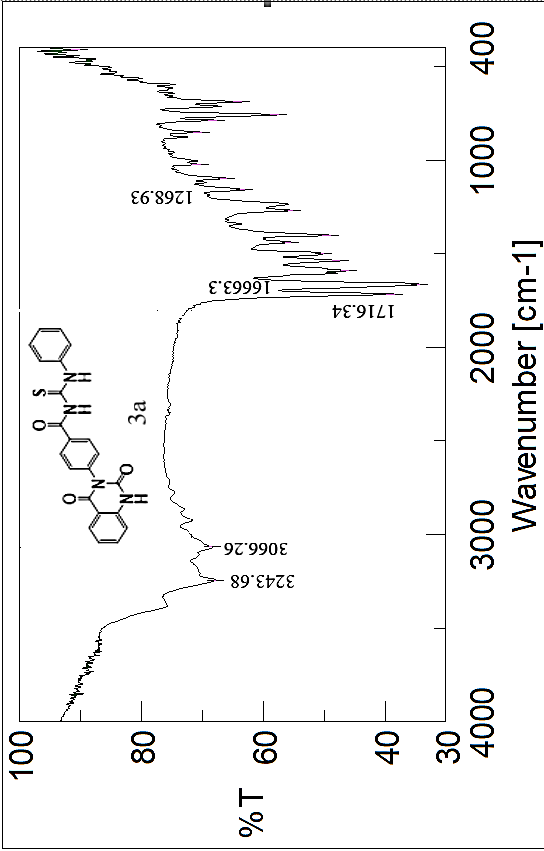


**Figure S9**: IR spectrum of compound **3a**.


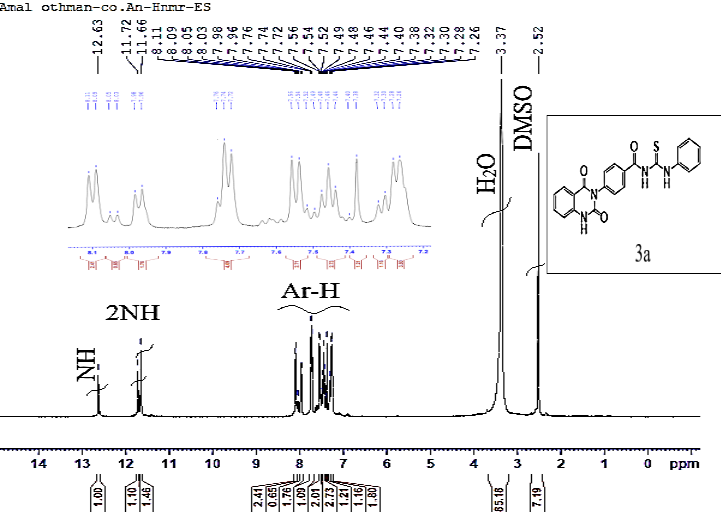


**Figure S10**: ^1^H-NMR spectrum of compound **3a**.


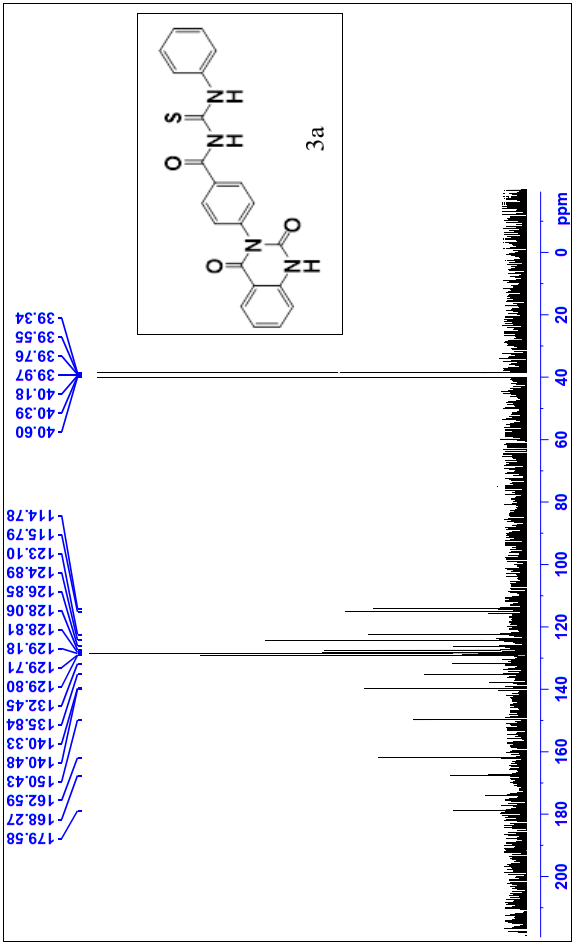


**Figure S11**: ^13^C-NMR spectrum of compound **3a**.


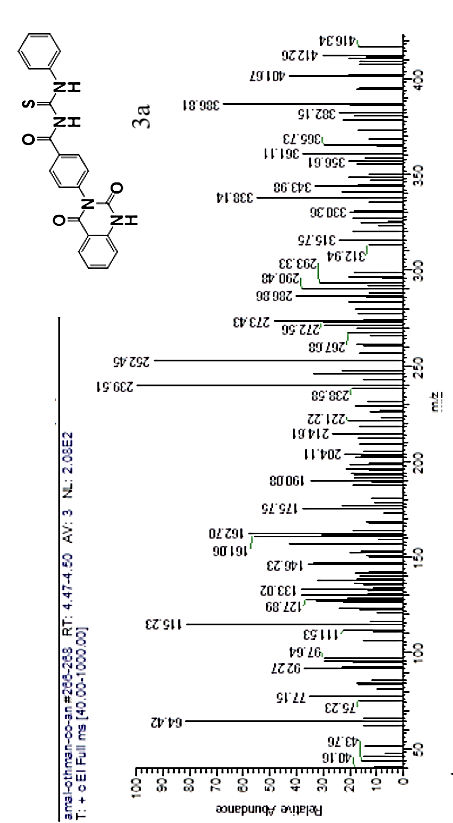


**Figure S12**: Mass spectrum of compound **3a**.


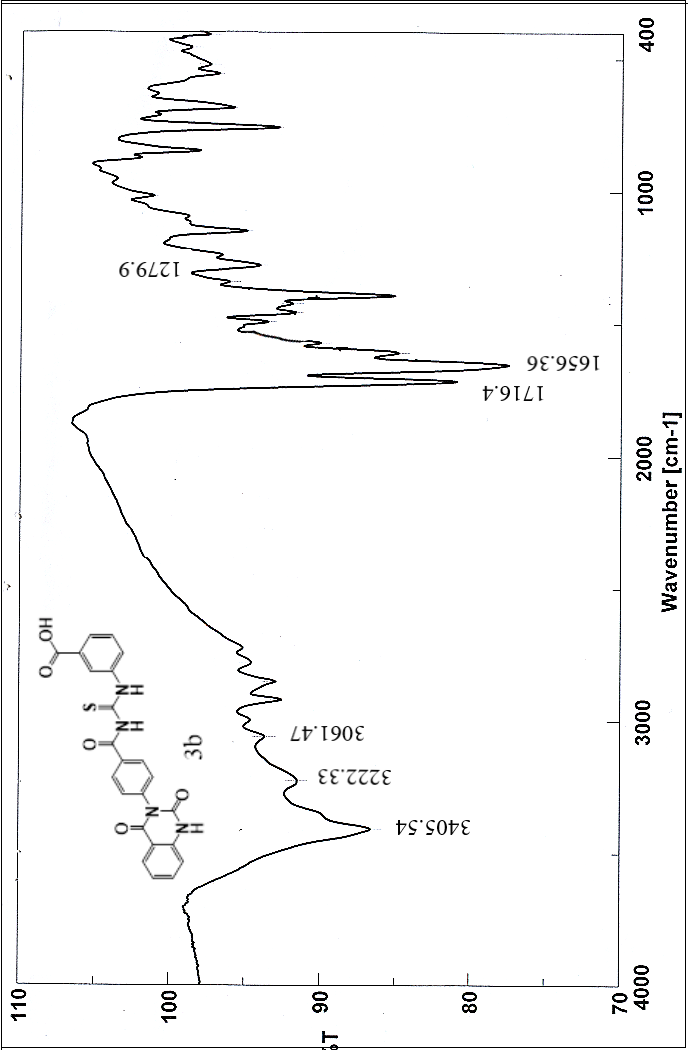


**Figure S13**: IR spectrum of compound **3b**.


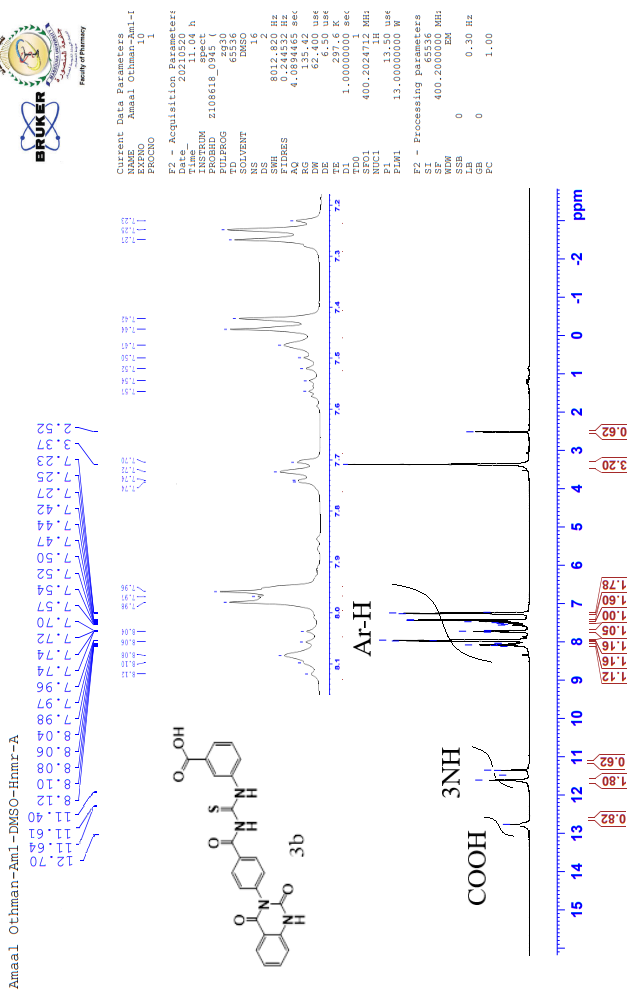


**Figure S14**: ^1^H-NMR spectrum of compound **3b**.


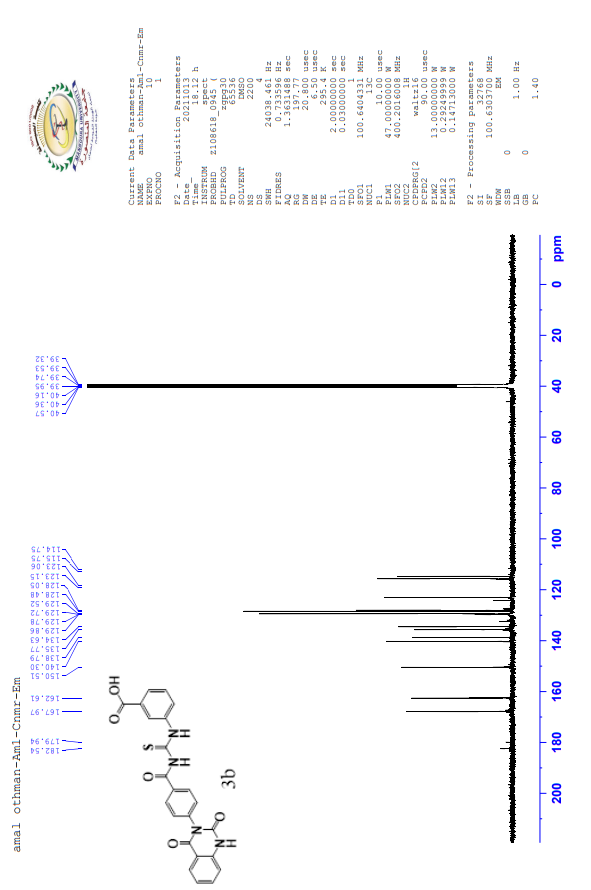


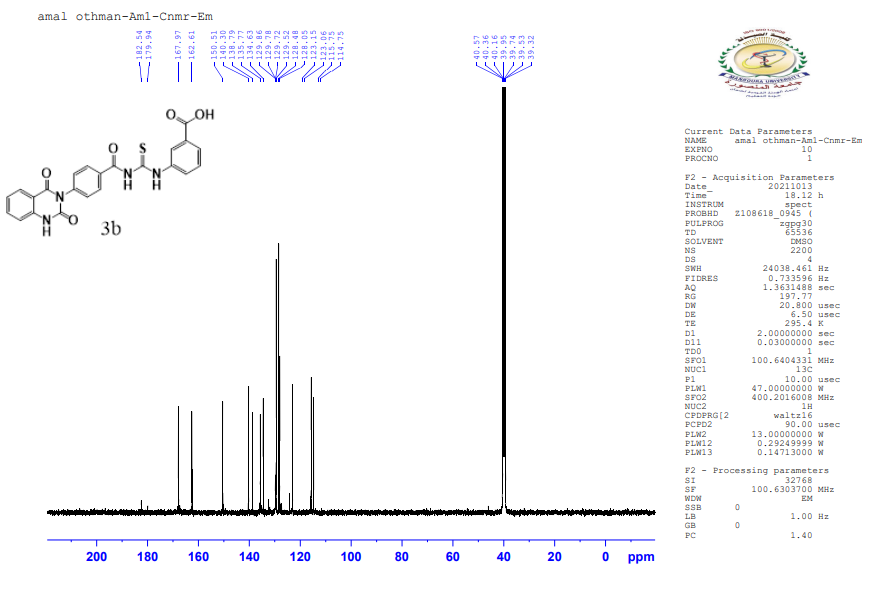


**Figure S15**: ^13^C-NMR spectrum of compound **3b**.


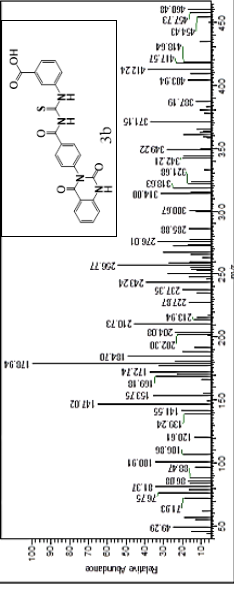


**Figure S16**: Mass spectrum of compound **3b**.


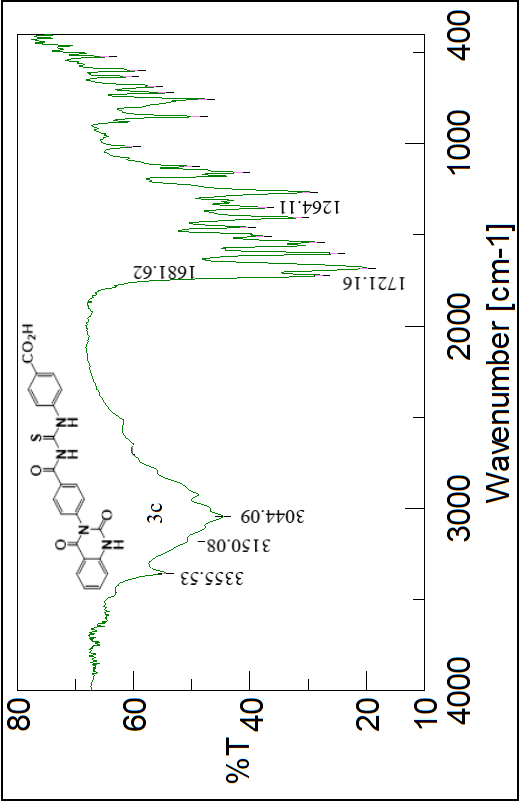


**Figure S17**: IR spectrum of compound **3c**.


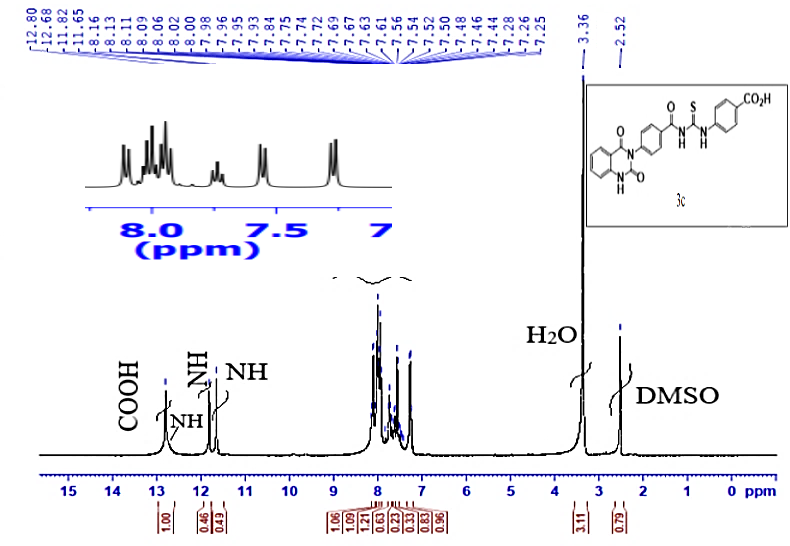


**Figure S18**: ^1^H-NMR spectrum of compound **3c**.


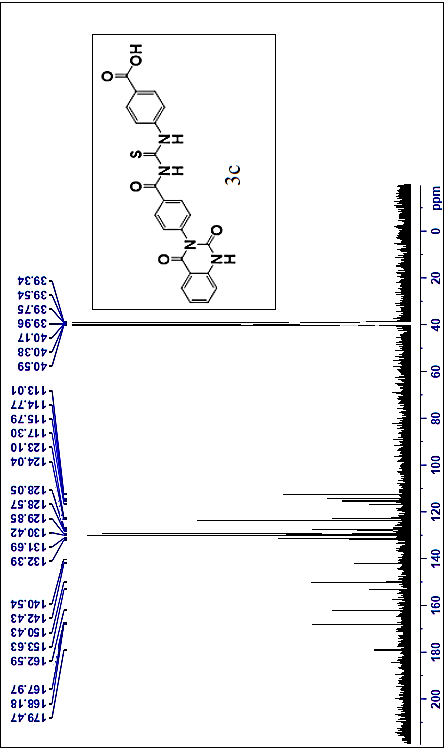


**Figure S19**: ^13^C-NMR spectrum of compound **3c**.


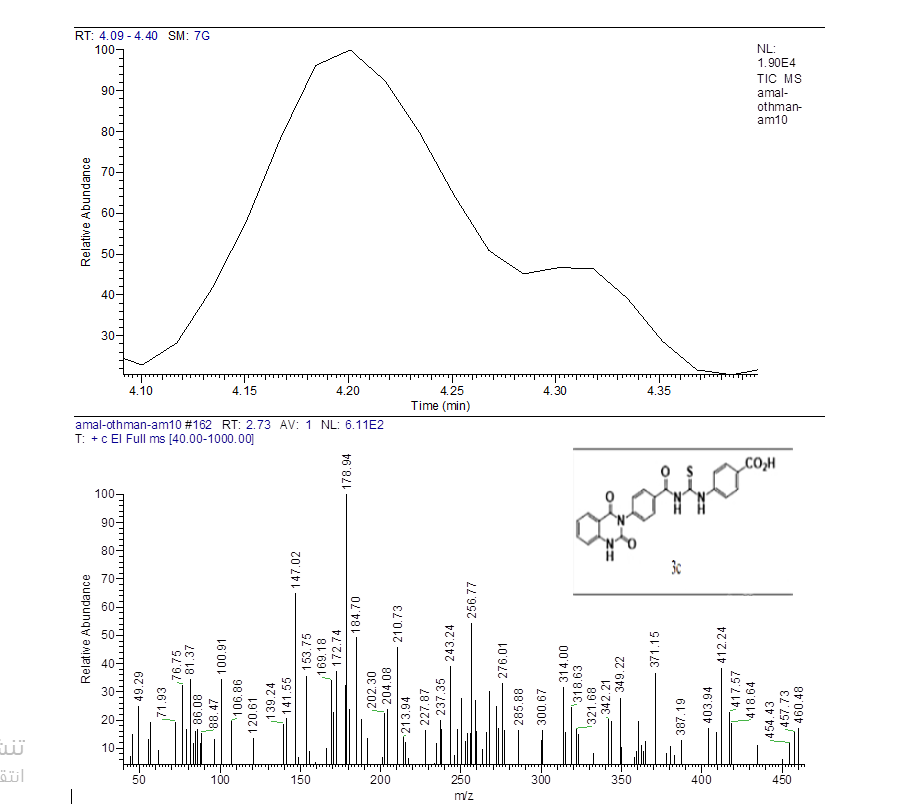


**Figure S20**: Mass spectrum of compound **3c**.


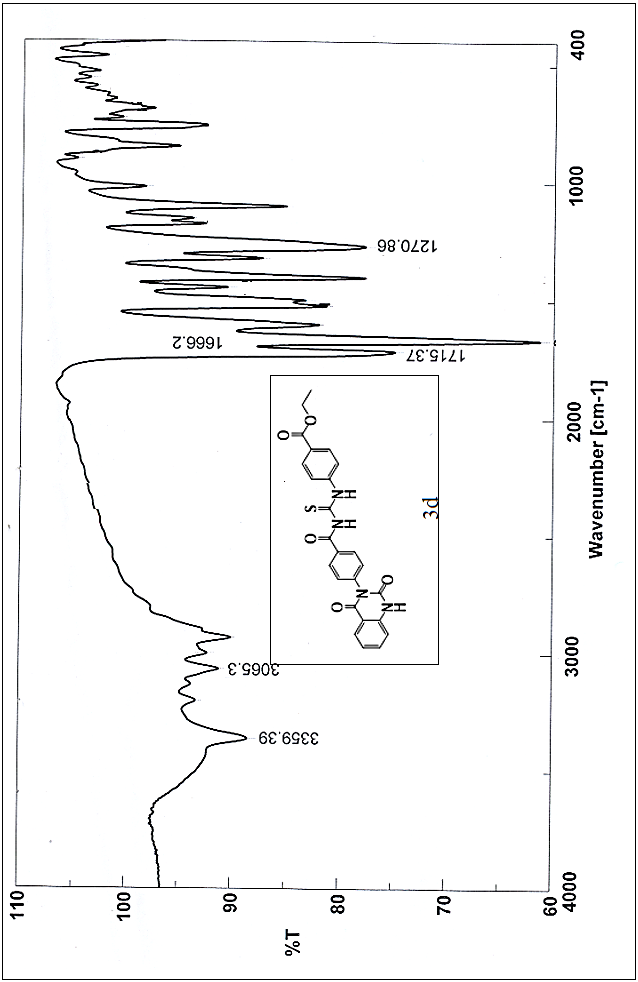


**Figure S21**: IR spectrum of compound **3d**.


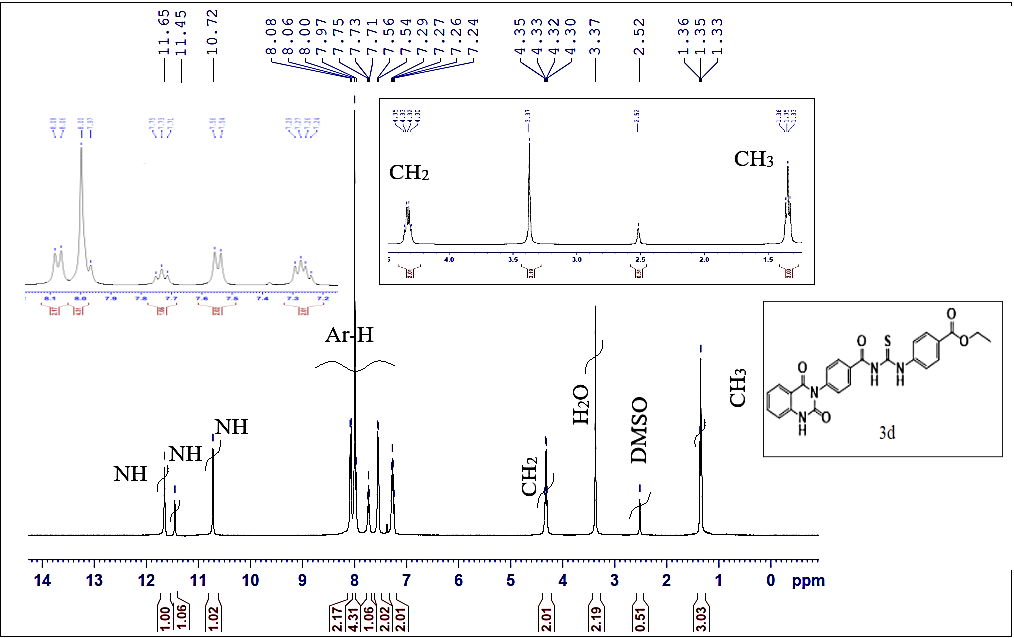


**Figure S22**: ^1^H-NMR spectrum of compound **3d**.


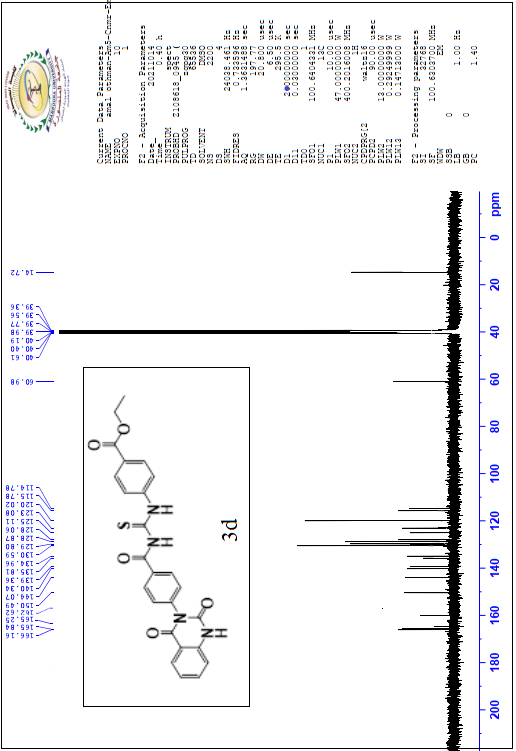


**Figure S23**: ^13^C-NMR spectrum of compound **3d**.


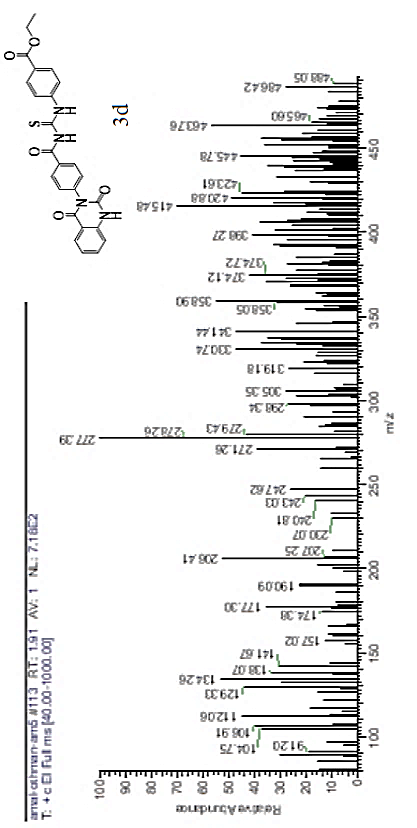


**Figure S24**: Mass spectrum of compound **3d**.


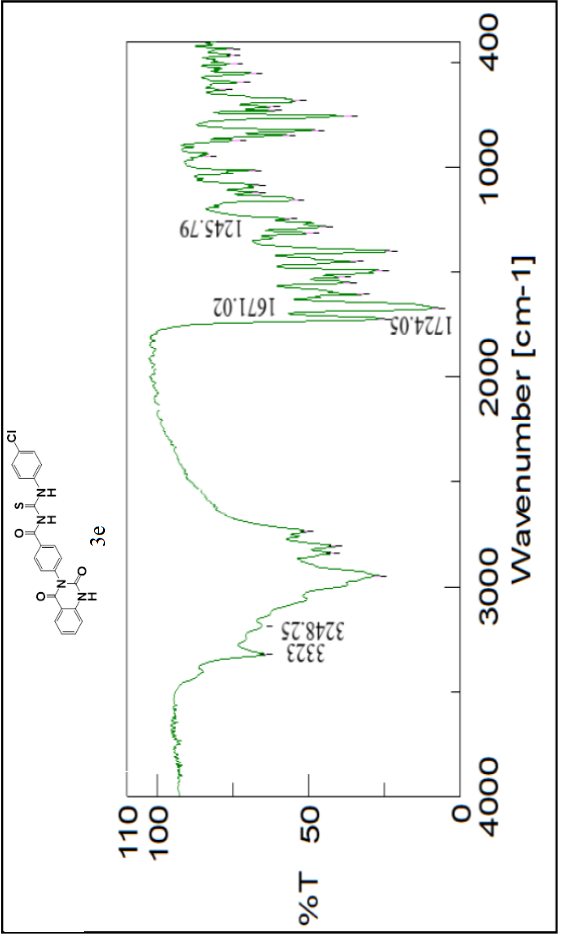


**Figure S25**: IR spectrum of compound **3e**.


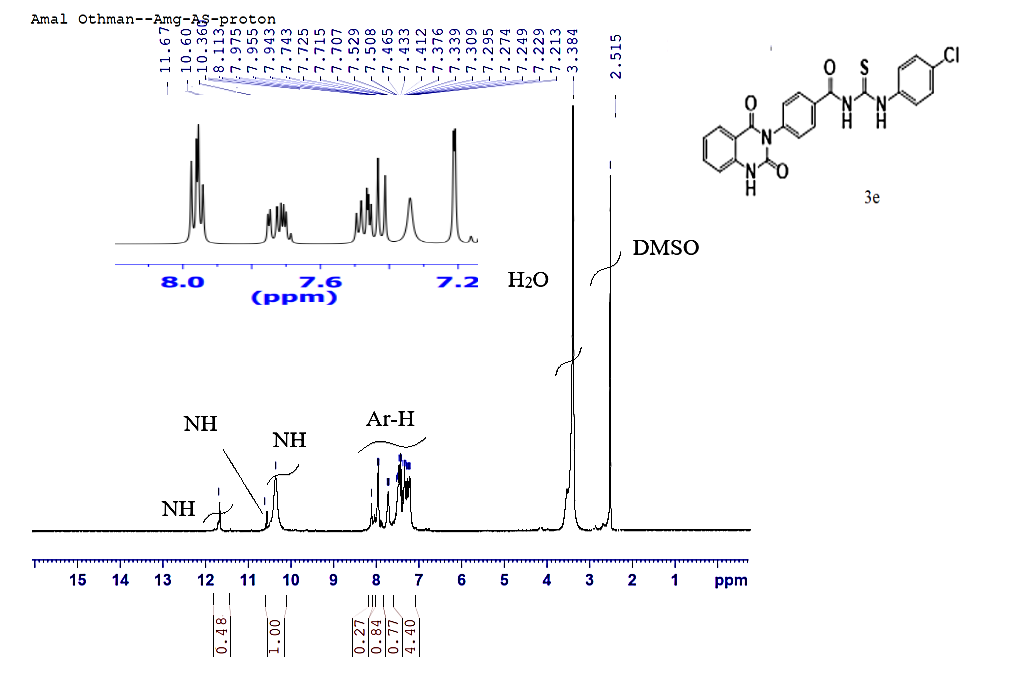


**Figure S26**: ^1^H-NMR spectrum of compound **3e**.


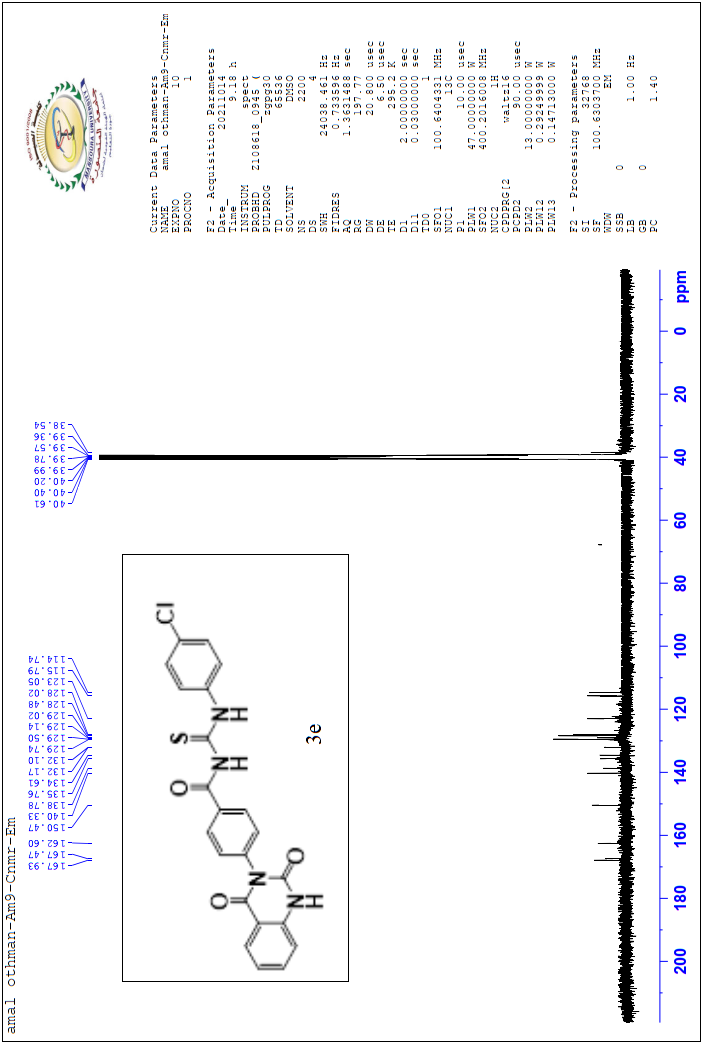
**Figure S27**: ^13^C-NMR spectrum of compound **3e**.


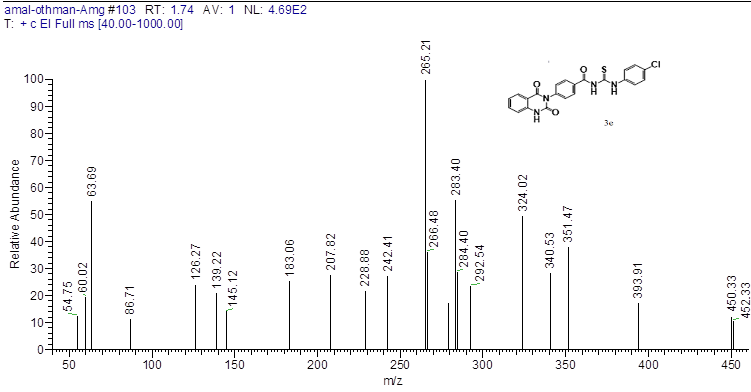


**Figure S28**: Mass spectrum of compound **3e**.


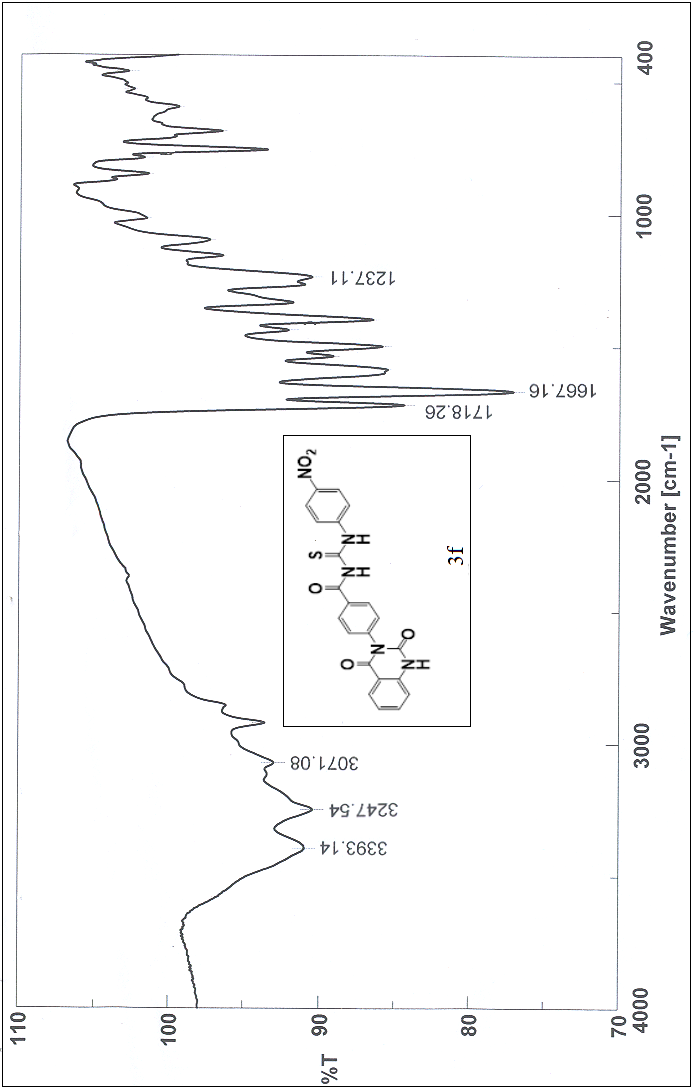


**Figure S29**: IR spectrum of compound **3f**.


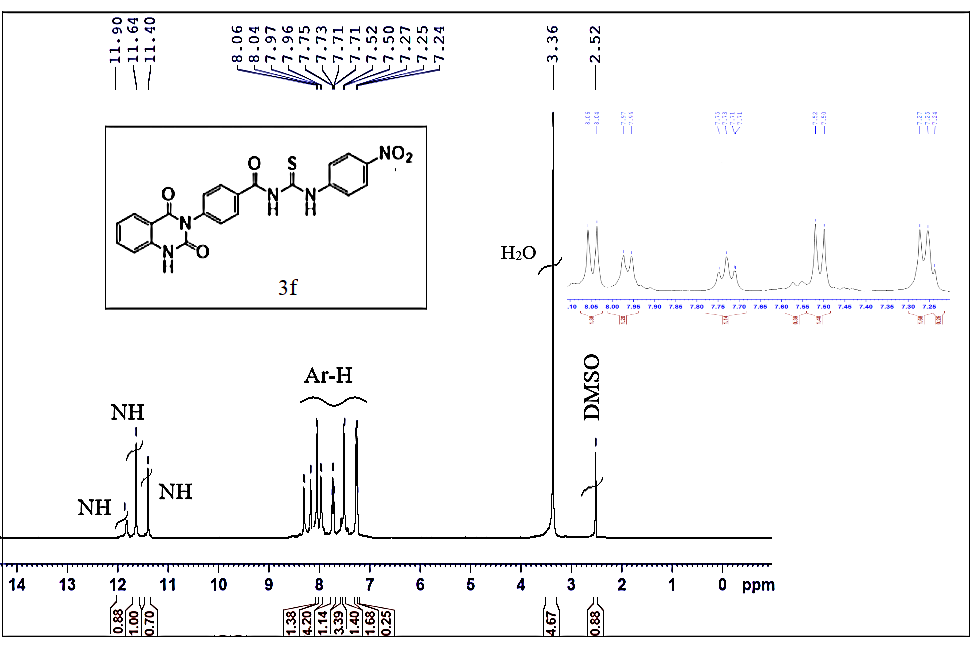


**Figure S30**: ^1^H-NMR spectrum of compound **3f**.


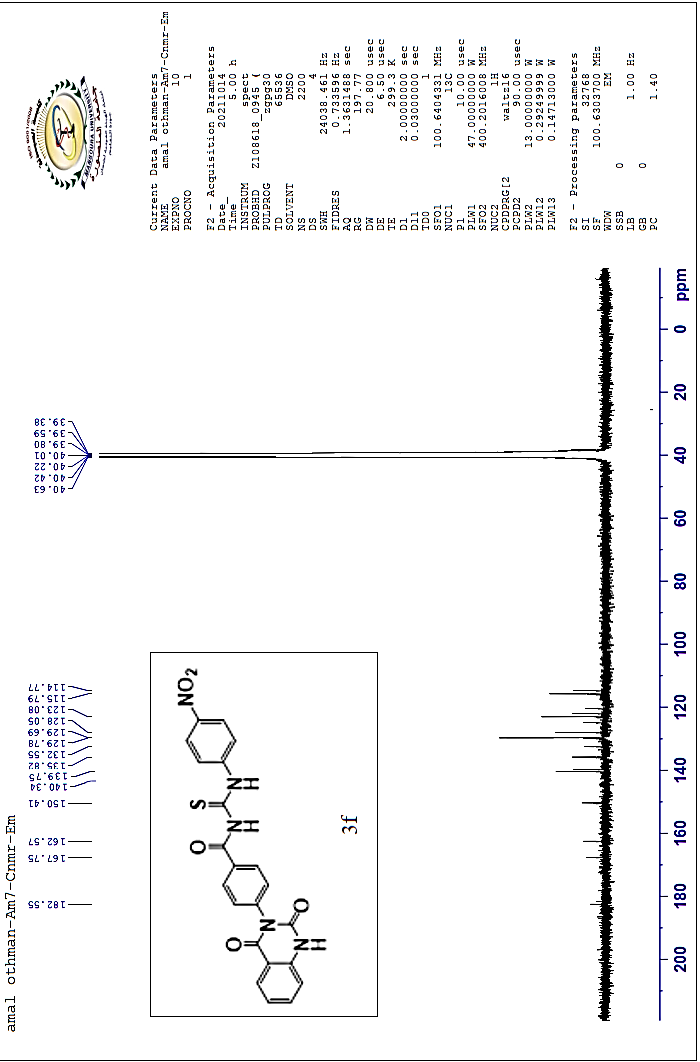


**Figure S31**: ^13^C-NMR spectrum of compound **3f**.


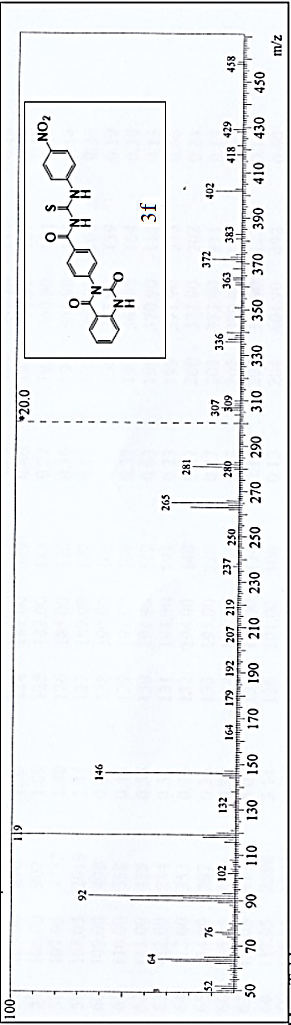


**Figure S32**: Mass spectrum of compound **3f**.


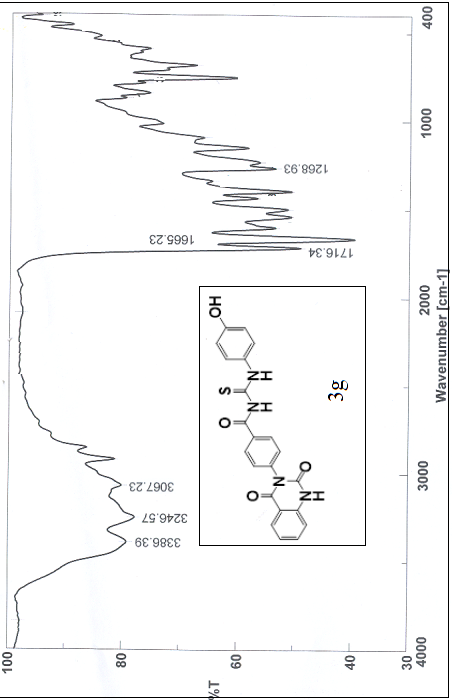


**Figure S33**: IR spectrum of compound **3g**.


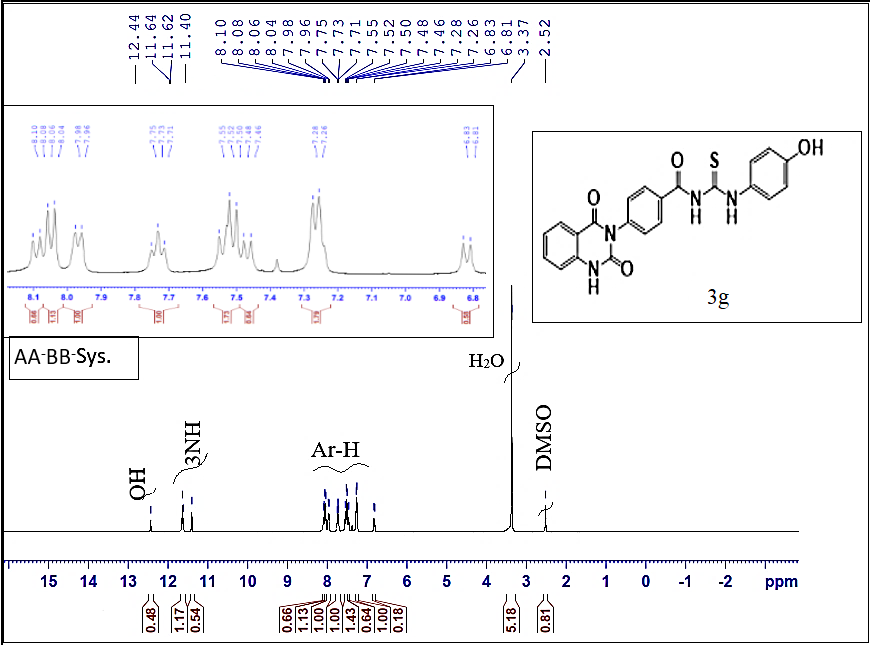


**Figure S34**: ^1^H-NMR spectrum of compound **3g**.


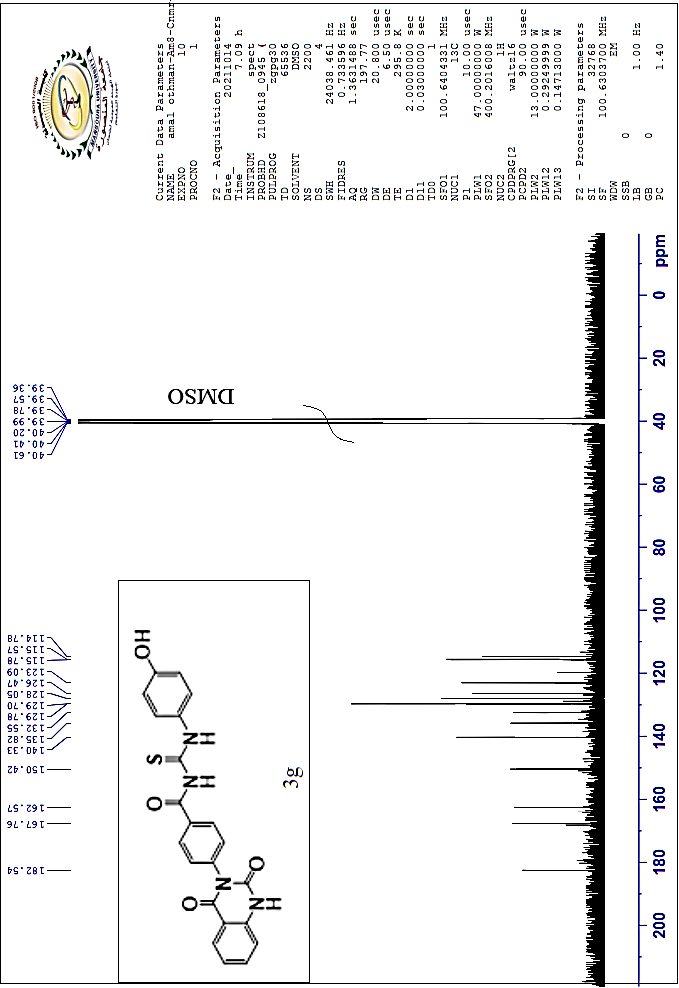


**Figure S35**: ^13^C-NMR spectrum of compound **3g**.


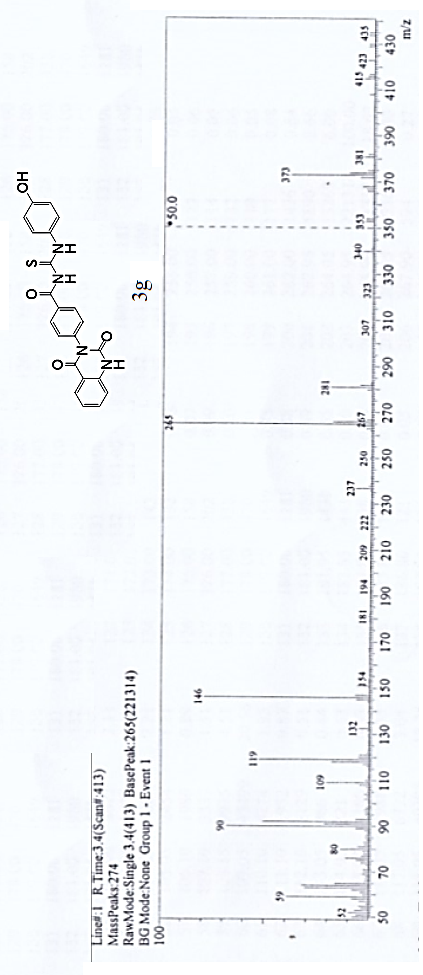


**Figure S36**: Mass spectrum of compound **3g**.


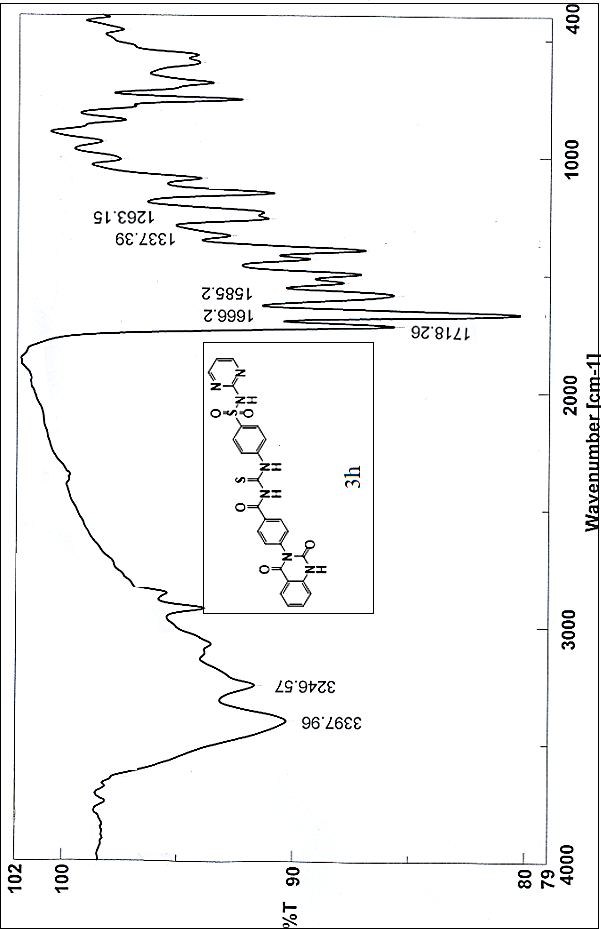


**Figure S37**: IR spectrum of compound **3h**.


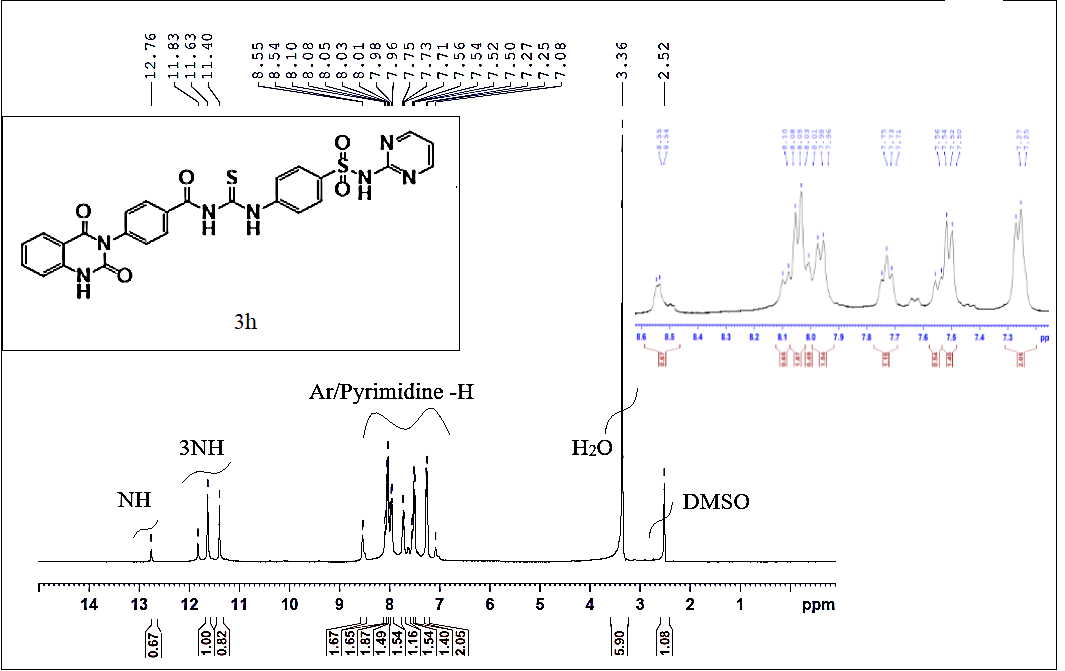


**Figure S38**: ^1^H-NMR spectrum of compound **3h**.


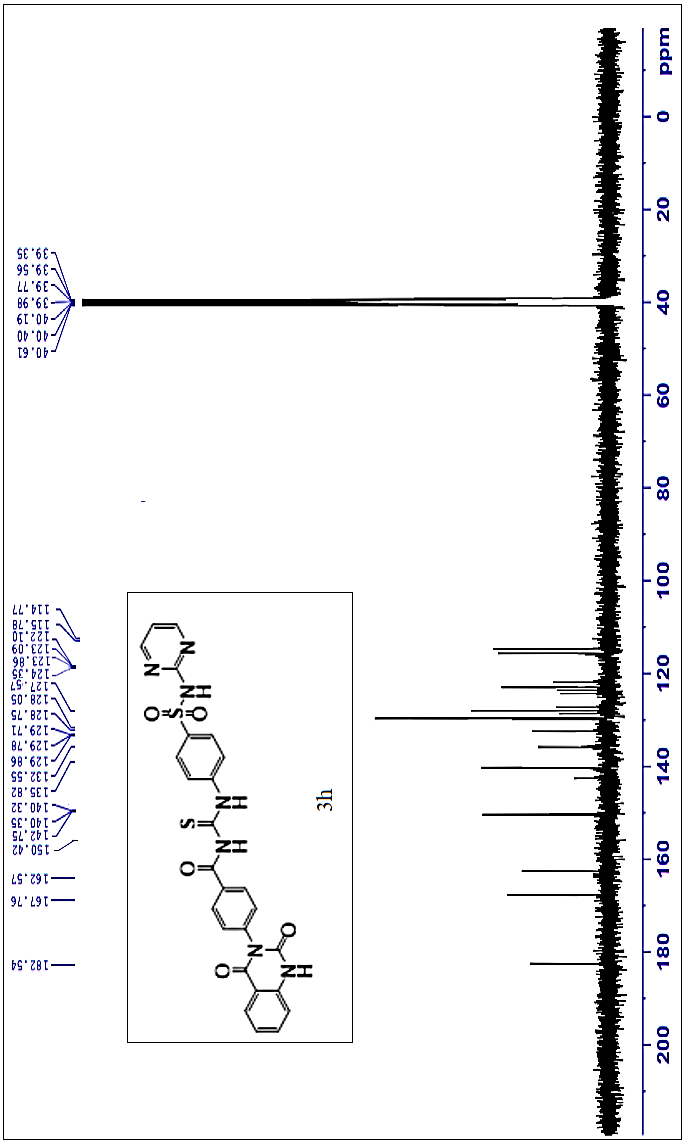


**Figure S39**: ^13^C-NMR spectrum of compound **3h**.


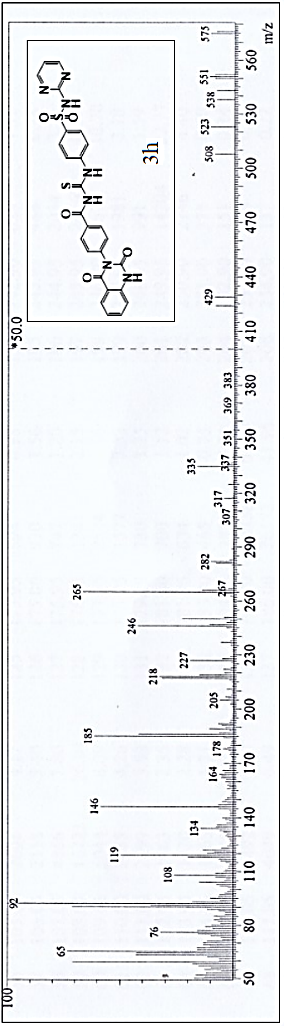


**Figure S40**: Mass spectrum of compound **3h**.


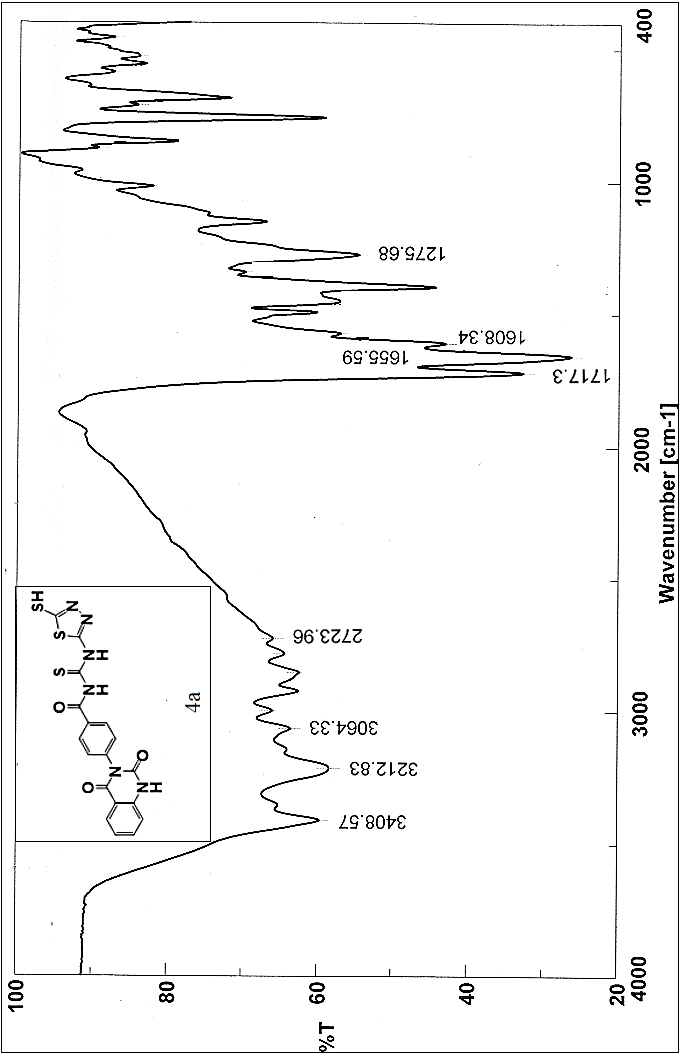


**Figure S41**: IR spectrum of compound **4a**.


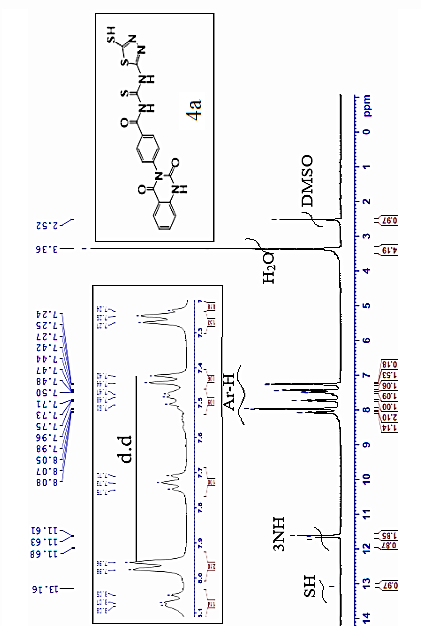


**Figure S42**: ^1^H-NMR spectrum of compound **4a**.


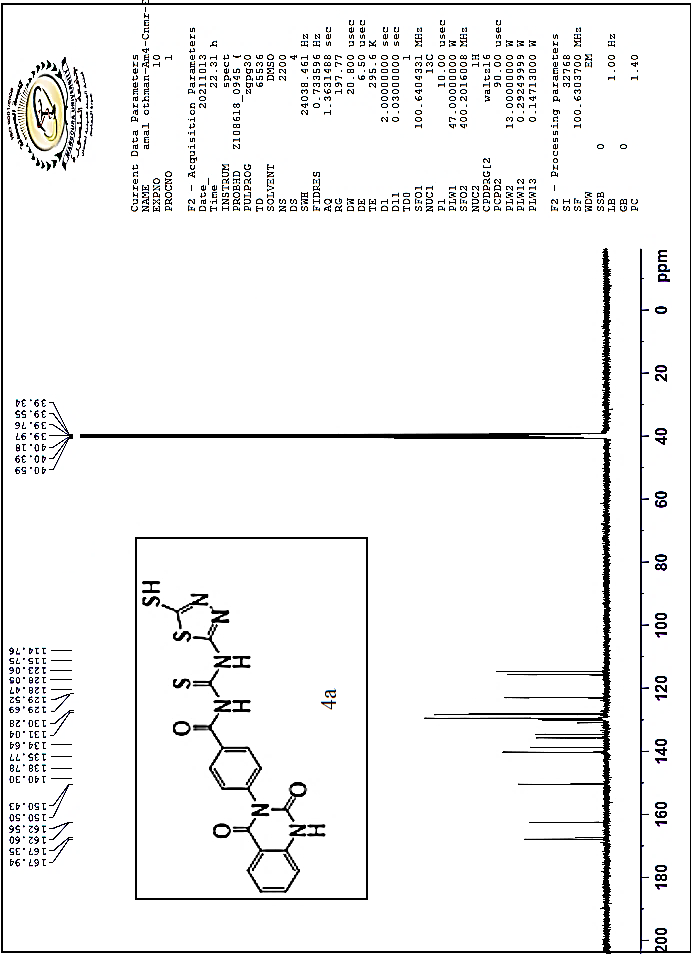


**Figure S43**: ^13^C-NMR spectrum of compound **4a**.


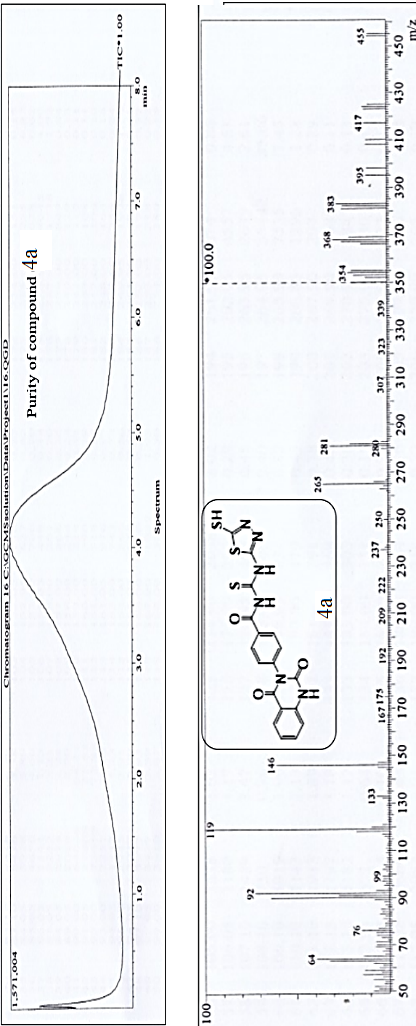


**Figure S44**: Mass spectrum of compound **4a**.


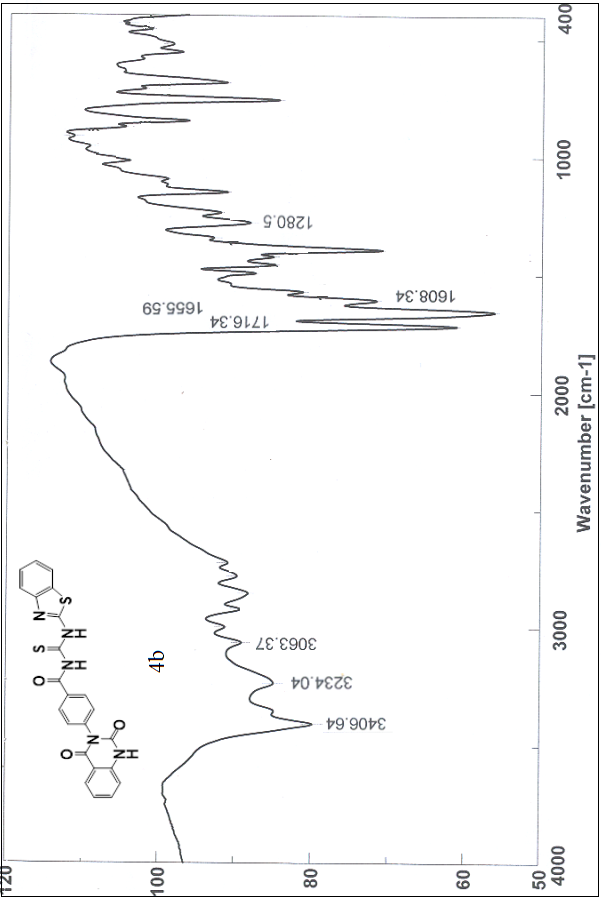


**Figure S45**: IR spectrum of compound **4b**.


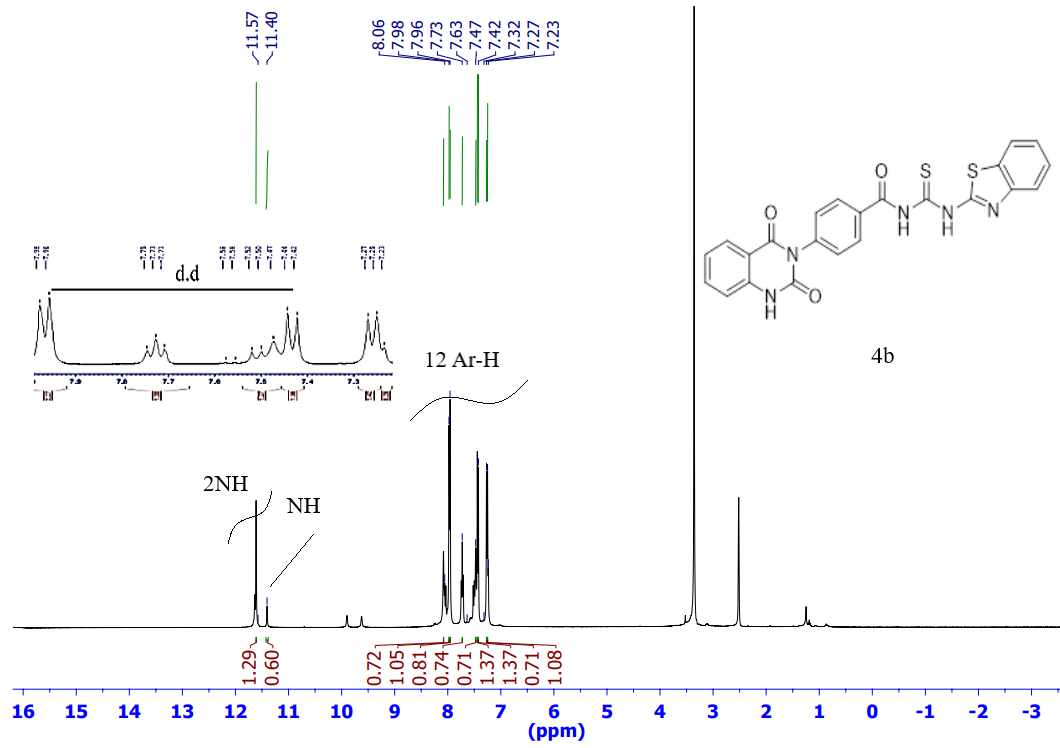


**Figure S46**: ^1^H-NMR spectrum of compound **4b**.


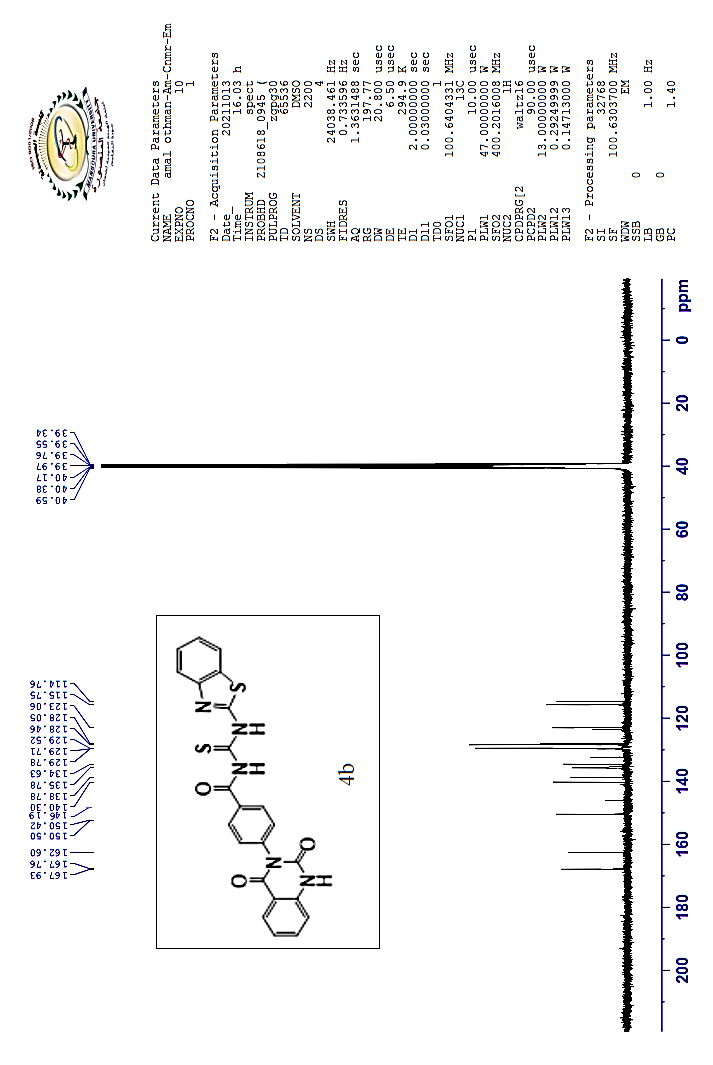


**Figure S47**: ^13^C-NMR spectrum of compound **4b**.


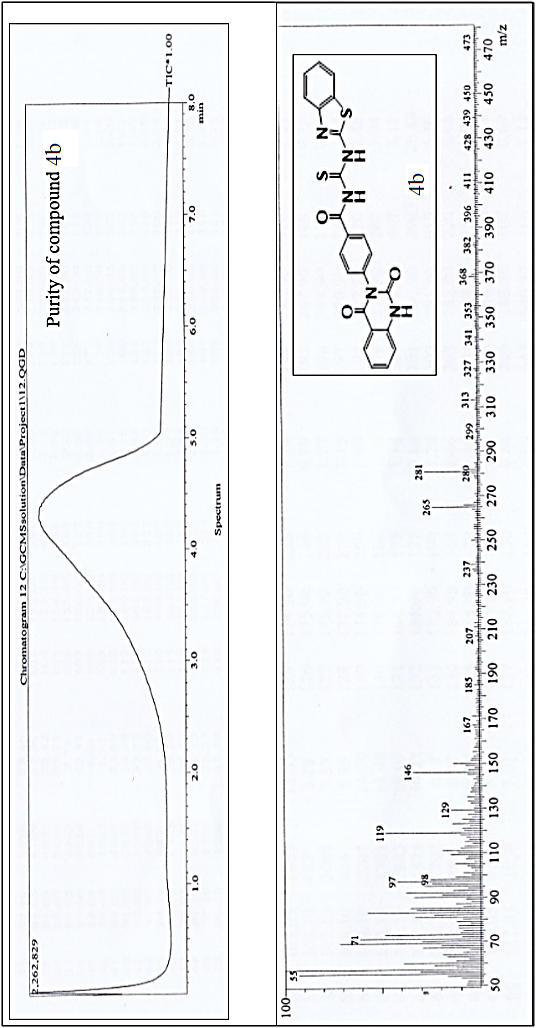


**Figure S48**: Mass spectrum of compound **4b**.


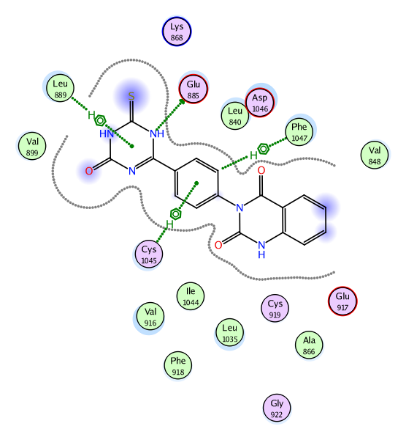


**Figure S49**: Docking style of compound **2a** with VEGFR-2 TK (PDB: 4asd).


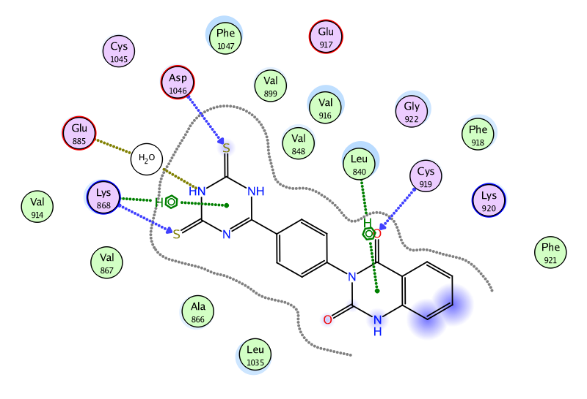


**Figure S50**: Docking style of compound **2b** with VEGFR-2 TK (PDB: 4asd).


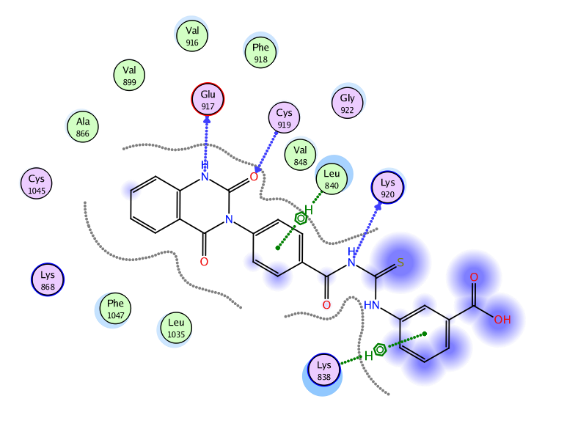


**Figure S51**: Docking style of compound **3b** with VEGFR-2 TK (PDB: 4asd).


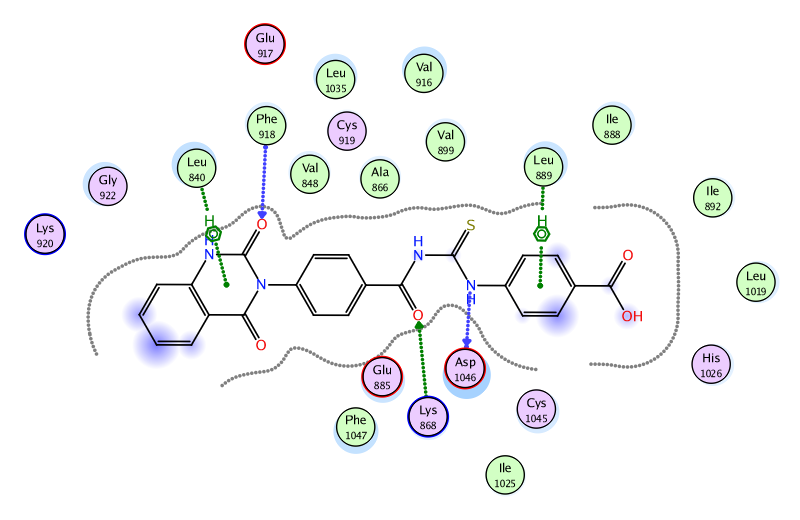


**Figure S52**: Docking style of compound **3c** with VEGFR-2 TK (PDB: 4asd).


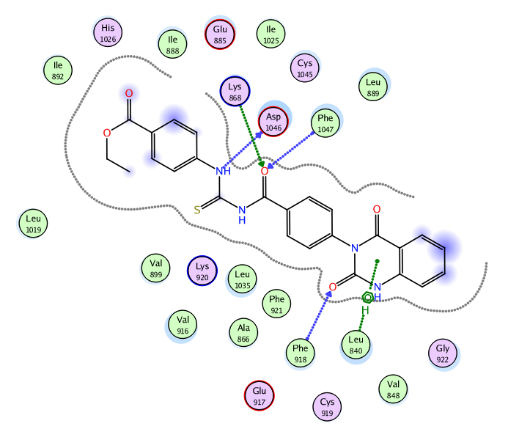


**Figure S53**: Docking style of compound **3d** with VEGFR-2 TK (PDB: 4asd).


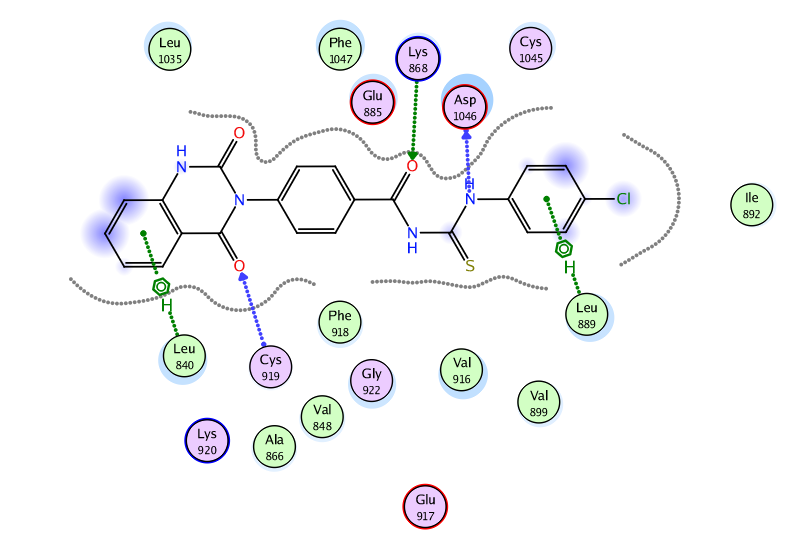


**Figure S54**: Docking style of compound **3e** with VEGFR-2 TK (PDB: 4asd).


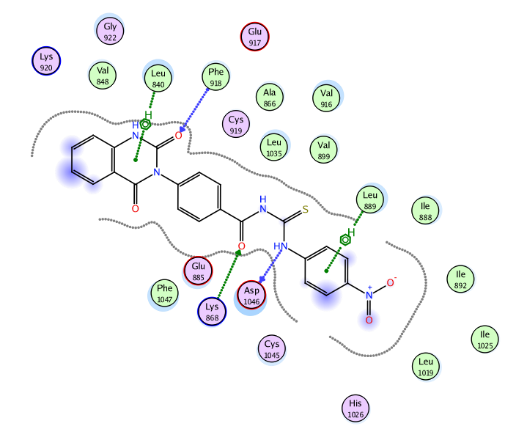


**Figure S55**: Docking style of compound **3f** with VEGFR-2 TK (PDB: 4asd).

**
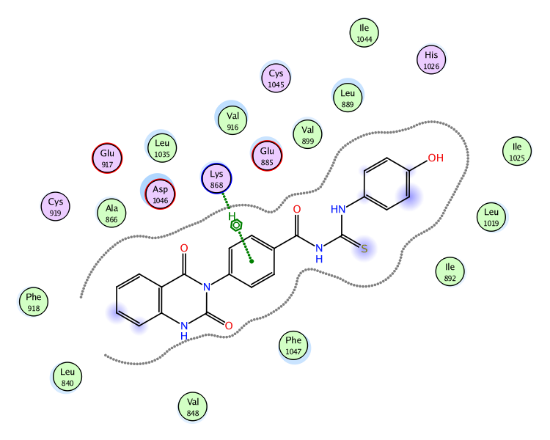
**

**Figure S56**: Docking style of compound **3g** with VEGFR-2 TK (PDB: 4asd).


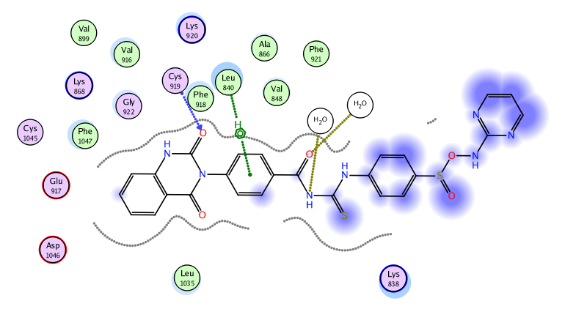


**Figure S57**: Docking style of compound **3h** with VEGFR-2 TK (PDB: 4asd).

**
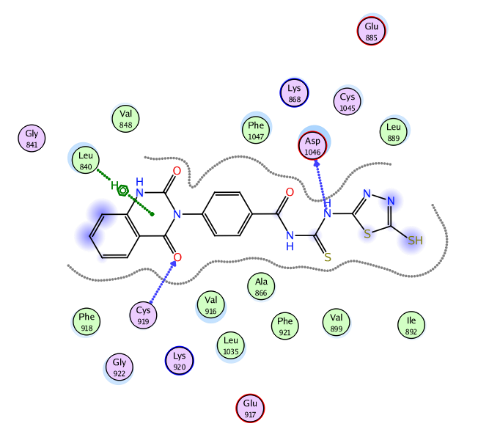
**

**Figure S58**: Docking style of compound **4a** with VEGFR-2 TK (PDB: 4asd).


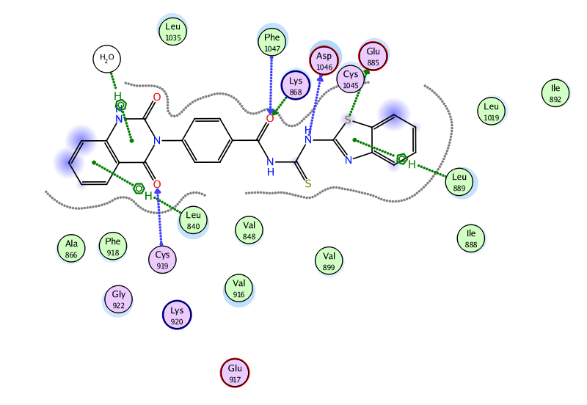


**Figure S59**: Docking style of compound **4b** with VEGFR-2 TK (PDB: 4asd).


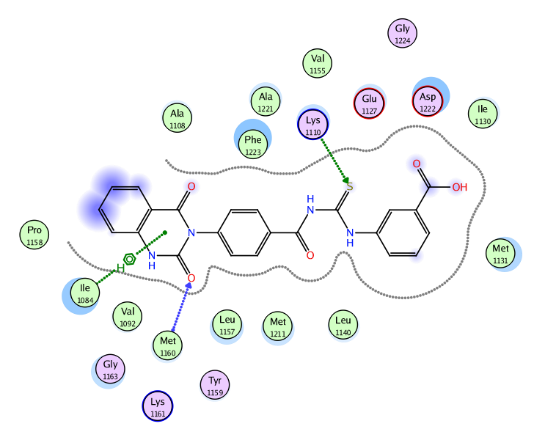


**Figure S60**: Docking style of compound **3b** with c-Met TK (PDB: 3lq8).


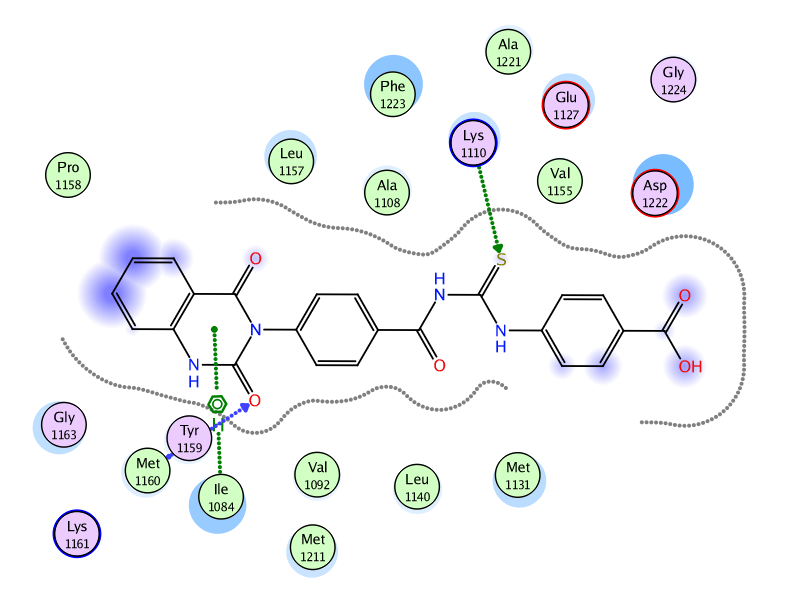


**Figure S61**: Docking style of compound **3c** with c-Met TK (PDB: 3lq8).


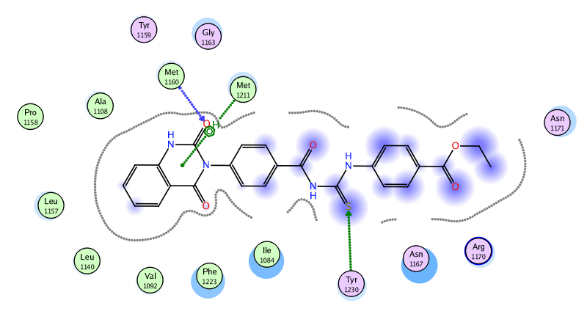


**Figure S62**: Docking style of compound **3d** with c-Met TK (PDB: 3lq8).


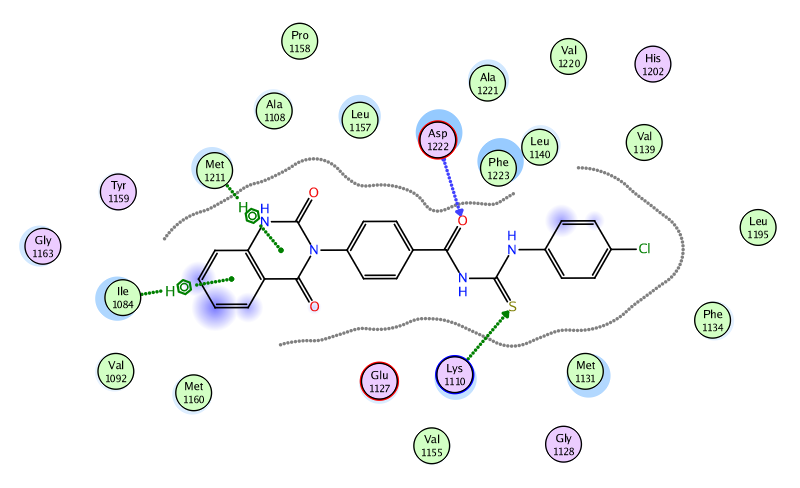


**Figure S63**: Docking style of compound **3e** with c-Met TK (PDB: 3lq8).


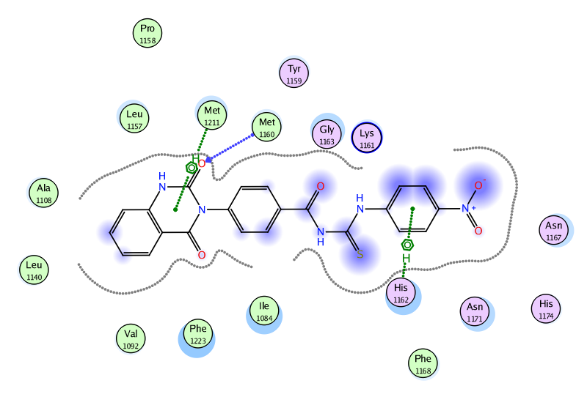


**Figure S64**: Docking style of compound **3f** with c-Met TK (PDB: 3lq8).


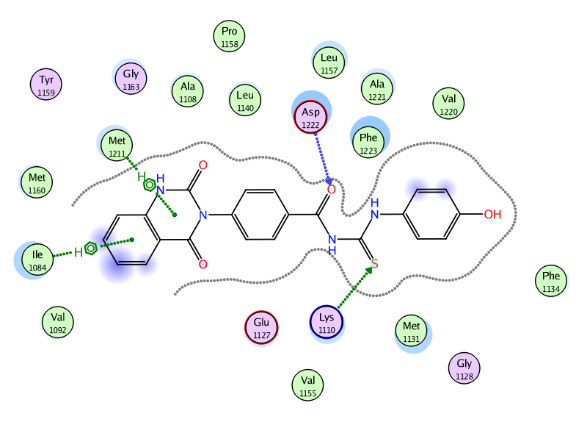


**Figure S65**: Docking style of compound **3g** with c-Met TK (PDB: 3lq8).


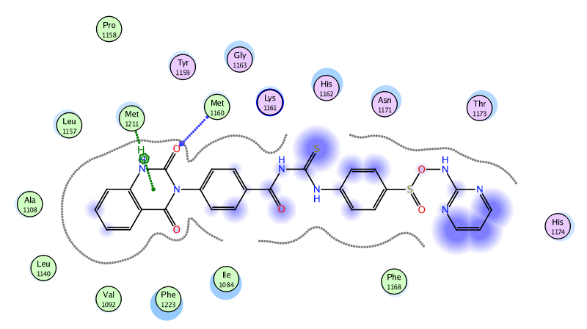


**Figure S66**: Docking style of compound **3h** with c-Met TK (PDB: 3lq8).


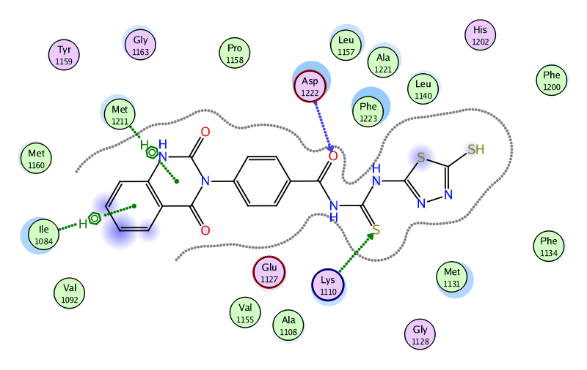


**Figure S67**: Docking style of compound **4a** with c-Met TK (PDB: 3lq8).


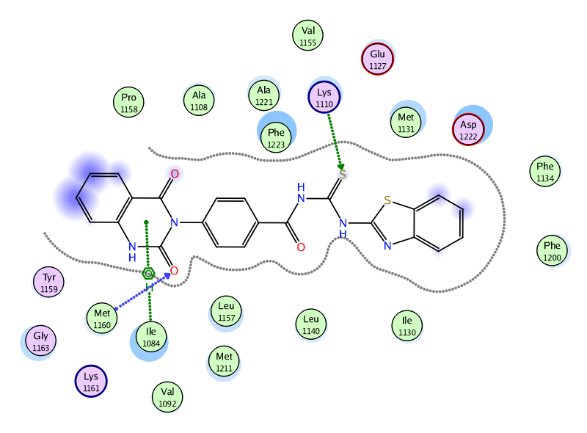


**Figure S68**: Docking style of compound **4b** with c-Met TK (PDB: 3lq8).

**Cell culture Protocol**

Cell Line cells were obtained from American Type Culture Collection , cells were cultured using DMEM (Invitrogen/Life Technologies) supplemented with 10% FBS (Hyclone), 10 ug/ml of insulin (Sigma), and 1% penicillin-streptomycin. All the other chemicals and reagents were from Sigma, or Invitrogen. Plate cells (cells density 1.2 – 1.8 × 10,000 cells/well) in a volume of 100µl complete growth medium + 100 μl of the tested compound per well in a 96-well plate for 24 hours before the MTT assay .

**Cell culture protocol**

1. Remove culture medium to a centrifuge tube.

2. Briefly rinse the cell layer with 0.25% (w/v) Trypsin 0.53 mM EDTA solution to remove all traces of serum which contains Trypsin inhibitor.

3. Add 2.0 to 3.0 ml of Trypsin EDTA solution to flask and observe cells under an inverted microscope until cell layer is dispersed (usually within 5 to 15 minutes).

Note: To avoid clumping do not agitate the cells by hitting or shaking the flask while waiting for the cells to detach. Cells that are difficult to detach may be placed at 37°C to facilitate dispersal.

4. Add 6.0 to 8.0 mL of complete growth medium and aspirate cells by gently pipetting.

5. Transfer the cell suspension to the centrifuge tube with the medium and cells from step 1, and centrifuge at approximately 125 xg for 5 to 10 minutes. Discard the supernatant.

6. Resuspend the cell pellet in fresh growth medium. Add appropriate aliquots of the cell suspension to new culture vessels.

7. Incubate cultures at 37°C for 24 hrs.

8-After treatment of cells with the serial concentrations of the compound to be tested incubation is carried out for 48 h at 37ºC ,then the plates are to be examined under the inverted microscope and proceed for the MTT assay

**MTT – Cytotoxicity assay protocol**

The MTT method of monitoring in vitro cytotoxicity is

well suited for use with multiwell plates. For best results, cells in the log phase of growth should be employed and final cell number should not exceed 106 cells/cm2. Each test should include a blank containing complete medium without cells.

1. Remove cultures from incubator into laminar flow hood or other sterile work area.

2. Reconstitute each vial of MTT [M-5655] to be used with 3 ml of medium or balanced salt solution without phenol red and serum. Add reconstituted MTT in an amount equal to 10% of the culture medium volume.

3. Return cultures to incubator for 2-4 hours depending on cell type and maximum cell density. (An incubation period of 2 hours is generally adequate but may be lengthened for low cell densities or cells with lower metabolic activity.) Incubation times should be consistent when making comparisons.

4. After the incubation period, remove cultures from incubator and dissolve the resulting formazan crystals by adding an amount of MTT Solubilization Solution [M-8910] equal to the original culture medium volume.

5. Gentle mixing in a gyratory shaker will enhance dissolution. Occasionally, especially in dense cultures, pipetting up and down [trituration] may be required to completely dissolve the MTT formazan crystals.

6. Spectrophotometrically measure absorbance at a wavelength of 570 nm. Measure the background absorbance of multiwell plates at 690 nm and subtract from the 450 nm measurement. Tests performed in multiwell plates can be read using the appropriate type of plate

reader or the contents of individual wells may be transferred to appropriate size cuvettes for spectrophotometric measurement.

***In silico prediction of physicochemical and pharmacokinetic properties***

The Swiss Institute of Bioinformatics provides a freely available tools for prediction of pharmacokinetics and physicochemical properties of small molecules via URL: [http://www.swissadme.ch/index.php#](http://www.swissadme.ch/index.php). Different filters were used to compute druglikeness as follow: Pfizer Lipinski (MW ≤ 500, MLOGP ≤ 4.15, N or O ≤ 10, NH or OH ≤ 5); Ghose (160 ≤ MW≤ 480, -0.4 ≤ WLOGP ≤ 5.6, 40 ≤ MR ≤ 130, 20 ≤ atoms ≤ 70); Veber (rotatable bonds ≤ 10, TPSA ≤ 140); Egan (WLOGP ≤ 5.88, TPSA ≤ 131.6); Muegge (200 ≤ MW≤ 600, -2 ≤ XLOGP ≤ 5, TPSA ≤ 150, rings ≤ 7, C > 4, heteroatoms > 1, rotatable bonds > 15, HBA ≤ 10, HBD ≤ 5); Abbott Bioavailability Score (probability of F > 10% in rat). iLOG. BOILED Egg is a plot of TPSA on the X axis versus WLOGP on the Y axis. The white oval is the suitable TPSA/WLOGP values for the highest probability of GI absorption, and the yolk circle is the suitable TPSA/WLOGP values for the highest probability to BBB accessibility. The optimal values of physicochemical properties involved in Bioavailability radar are as follow: size (150 g/mol < MV > 500 g/mol), polarity (20 Å2 <TPSA > 130 Å2), lipophilicity (-0.7 < XLOGP3 > +5.0), insolubility (0 < Log S (ESOL) > 6), insaturation (0.25 < Fraction Csp3 > 1.0), and flexibility (rotatable bonds < 9).
